# Long-term Host Immune Response Trajectories Among Hospitalized Patients With Sepsis

**DOI:** 10.1001/jamanetworkopen.2019.8686

**Published:** 2019-08-07

**Authors:** Sachin Yende, John A. Kellum, Victor B. Talisa, Octavia M. Peck Palmer, Chung-Chou H. Chang, Michael R. Filbin, Nathan I. Shapiro, Peter C. Hou, Arvind Venkat, Frank LoVecchio, Katrina Hawkins, Elliott D. Crouser, Anne B. Newman, Derek C. Angus

**Affiliations:** 1 Veterans Affairs Pittsburgh Healthcare System, Pittsburgh, Pennsylvania; 2 Clinical Research, Investigation, and Systems Modeling of Acute Illness Center, University of Pittsburgh, Pittsburgh, Pennsylvania; 3 Center for Critical Care Nephrology, Department of Critical Care Medicine, University of Pittsburgh, Pittsburgh, Pennsylvania; 4 Department of Pathology, University of Pittsburgh School of Medicine, Pittsburgh, Pennsylvania; 5 Department of Medicine, University of Pittsburgh, Pittsburgh, Pennsylvania; 6 Department of Emergency Medicine, Massachusetts General Hospital, Boston; 7 Department of Emergency Medicine, Beth Israel Deaconess Medical Center, Boston, Massachusetts; 8 Division of Emergency Critical Care Medicine, Department of Emergency Medicine, Brigham and Women’s Hospital, Boston, Massachusetts; 9 Department of Emergency Medicine, Allegheny Health Network, Pittsburgh, Pennsylvania; 10 Maricopa Medical Center, Phoenix, Arizona; 11 Department of Anesthesia, George Washington University School of Medicine & Health Sciences, Washington, DC; 12 Department of Medicine, George Washington University School of Medicine & Health Sciences, Washington, DC; 13 Division of Pulmonary, Critical Care and Sleep Medicine, The Ohio State University Wexner Medical Center, Columbus; 14 Department of Epidemiology, University of Pittsburgh, Graduate School of Public Health, and School of Medicine, Division of Geriatric Medicine, Pittsburgh, Pennsylvania

## Abstract

**Importance:**

Long-term immune sequelae after sepsis are poorly understood.

**Objective:**

To assess whether abnormalities in the host immune response during hospitalization for sepsis persist after discharge.

**Design, Settings, and Participants:**

This prospective, multicenter cohort study enrolled and followed up for 1 year adults who survived a hospitalization for sepsis from January 10, 2012, to May 25, 2017, at 12 US hospitals.

**Exposures:**

Circulating levels of inflammation (interleukin 6 and high-sensitivity C-reactive protein [hs-CRP]), immunosuppression (soluble programmed death ligand 1 [sPD-L1]), hemostasis (plasminogen activator inhibitor 1 and D-dimer), endothelial dysfunction (E-selectin, intercellular adhesion molecule 1, and vascular cell adhesion molecule 1), and oxidative stress biomarkers were measured at 5 time points during and after hospitalization for sepsis for 1 year. Individual biomarker trajectories and patterns of trajectories across biomarkers (phenotypes) were identified.

**Main Outcomes and Measures:**

Outcomes were adjudicated centrally and included all-cause and cause-specific readmissions and mortality.

**Results:**

A total of 483 patients (mean [SD] age, 60.5 [15.2] years; 265 [54.9%] male) who survived hospitalization for sepsis were included in the study. A total of 376 patients (77.8%) had at least 1 chronic disease, and their mean (SD) Sequential Organ Failure Assessment score was 4.2 (3.0). Readmissions were common (485 readmissions in 205 patients [42.5%]), and 43 patients (8.9%) died by 3 months, 56 patients (11.6%) died by 6 months, and 85 patients (17.6%) died by 12 months. Elevated hs-CRP levels were observed in 23 patients (25.8%) at 3 months, 26 patients (30.2%) at 6 months, and 23 patients (25.6%) at 12 months, and elevated sPD-L1 levels were observed in 45 patients (46.4%) at 3 months, 40 patients (44.9%) at 6 months, and 44 patients (49.4%) at 12 months. Two common phenotypes were identified based on hs-CRP and sPDL1 trajectories: high hs-CRP and sPDL1 levels (hyperinflammation and immunosuppression phenotype [326 of 477 (68.3%)]) and normal hs-CRP and sPDL1 levels (normal phenotype [143 of 477 (30.0%)]). These phenotypes had similar clinical characteristics and clinical course during hospitalization for sepsis. Compared with normal phenotype, those with the hyperinflammation and immunosuppression phenotype had higher 1-year mortality (odds ratio, 8.26; 95% CI, 3.45-21.69; *P* < .001), 6-month all-cause readmission or mortality (hazard ratio [HR], 1.53; 95% CI, 1.10-2.13; *P* = .01), and 6-month readmission or mortality attributable to cardiovascular disease (HR, 5.07; 95% CI, 1.18-21.84; *P* = .02) or cancer (HR, 5.15; 95% CI, 1.25-21.18; *P* = .02). These associations were adjusted for demographic characteristics, chronic diseases, illness severity, organ support, and infection site during sepsis hospitalization and were robust in sensitivity analyses.

**Conclusions and Relevance:**

In this study, persistent elevation of inflammation and immunosuppression biomarkers occurred in two-thirds of patients who survived a hospitalization for sepsis and was associated with worse long-term outcomes.

## Key Points

**Question:**

Does sepsis have immune sequelae, and are these sequelae associated with poor long-term outcomes?

**Findings:**

In this cohort study of 483 patients who survived hospitalization with sepsis at 12 US hospitals, 25.8% had elevated high-sensitivity C-reactive protein levels (a marker of inflammation) at 3 months, 30.2% at 6 months, and 25.6% at 12 months and 46.4% had elevated soluble programmed death ligand 1 levels (a marker of immunosuppression) at 3 months, 44.9% at 6 months, and 49.4% at 12 months. Two common phenotypes were identified based on these markers, and patients with hyperinflammation and immunosuppression had a higher risk of readmission or death.

**Meaning:**

Persistent elevation of inflammation and immunosuppression markers is common up to a year in patients who survive hospitalization for sepsis and may be associated with poor long-term outcomes.

## Teaser

This multicenter cohort study of adults who have survived hospitalization for sepsis assesses whether abnormalities in the host immune response during hospitalization for sepsis persist after discharge.

## Introduction

Sepsis is defined as life-threatening organ dysfunction caused by a dysregulated host immune response to infection.^1^ Long-term sequelae are common after an episode of sepsis,^2^ and patients who survive a hospitalization for sepsis have high 1-year mortality.^3^ Survivors are frequently readmitted and have physical and cognitive deficits and poor quality of life.^2,4,5^ Prior studies^6,7^ have found that these sequelae cannot be solely attributed to poor health status before sepsis admission. However, the underlying mechanisms remain poorly understood.

Persistent dysregulation of the host immune response after hospital discharge may contribute to long-term sequelae after sepsis.^2^ Evidence that supports this theory includes demonstration in a clinical study^6^ that elevations in circulating interleukin (IL) 6 and IL-10 concentrations persist at hospital discharge and are associated with late sequelae. In animal models, similar elevations persisted for several months after experimental sepsis compared with sham treatment.^8^ Whether immune response abnormalities persist after hospital discharge and increase the risk of long-term sequelae in humans is not known. If dysregulated immune response persists, there may be a need to test long-term immunomodulatory strategies in sepsis survivors.

We sought to characterize circulating biomarker trajectories in 5 domains (inflammation, immunosuppression, hemostasis, endothelial dysfunction, and oxidative stress) during 1 year in patients hospitalized with sepsis. We tested the hypotheses that biomarker trajectories or phenotypes that suggest persistent immune dysregulation were associated with higher risk for mortality and readmissions.

## Methods

### Patients and Design

We conducted a prospective cohort study of patients hospitalized with sepsis at 12 US sites from January 10, 2012, to May 25, 2017 (eMethods in the Supplement). We included adults who met criteria for sepsis (suspected infection with at least 1 organ dysfunction and similar to the recent Sepsis-3 criteria^1^) and enrolled them within 72 hours of hospital admission. Detailed eligibility criteria are included in the eMethods in the Supplement. We obtained approval from the institutional review boards of the University of Pittsburgh and all participating sites. Patients or their surrogates provided written informed consent. We obtained patient identifiers from the sites to allow us to contact the patient for long-term follow-up. This study followed the Strengthening the Reporting of Observational Studies in Epidemiology (STROBE) reporting guideline.

### Study Procedures

#### Index Hospitalization for Sepsis

After enrollment, we gathered detailed baseline and sequential clinical information using structured patient or surrogate interviews, bedside assessments, and medical record abstraction. We obtained a blood sample within hours of enrollment and 7 to 11 days after enrollment if the patient remained hospitalized.

#### Home Visits

We contacted patients by telephone and visited their home at 3, 6, and 12 months after enrollment to conduct structured interviews and obtain a blood sample. We ensured follow-up by obtaining contact information for the patient and 2 individuals who did not reside in the same household and by contacting them in the evening and on weekends if we failed to contact the patient during the day. Because we enrolled patients in 8 US states, we used a national home health agency to conduct home visits. We agreed to conduct the home visits at a convenient time and offered monetary compensation. We did not conduct home visits if patients were readmitted within the preceding 6 weeks because higher biomarker levels could be attributable to the readmission rather than the initial admission for sepsis. We did not conduct visits if patients were in a long-term care facility because they often have subclinical infections and because they are considered to be vulnerable individuals requiring additional regulatory approval from the long-term care facility and department of health in each state.

### Outcomes

We ascertained planned and unplanned readmissions during patient or next-of-kin interviews at 3, 6, and 12 months and queried the health records of the health care system where the patients were enrolled (eMethods in the Supplement). We centrally adjudicated causes for each readmission using a structured approach.^9^ Two reviewers determined the cause of readmission for a subset (n = 126), and the Cohen κ showed good agreement between the raters (κ = 0.79, *P* < .001). We determined whether patients had died and adjudicated the cause of death using telephone follow-up, review of hospitalization records, and National Death Index search (eMethods in the Supplement).

### Laboratory Procedures

We assessed the host immune response by measuring biomarkers at 5 time points during the index hospitalization (0-72 hours and 7-11 days) and at home (3, 6, and 12 months). At each time point, we measured biomarkers of inflammation (IL-6 and high-sensitivity C-reactive protein [hs-CRP]), immunosuppression (soluble programmed death ligand 1 [sPD-L1]), hemostasis (plasminogen activator inhibitor 1 and D-dimer), endothelial dysfunction (E-selectin, intercellular adhesion molecule 1, and vascular cell adhesion molecule 1), and oxidative stress (nitrate). We selected these pathways because they are activated during sepsis and may lead to readmissions and death due to cardiovascular disease, infection, and cancer in sepsis survivors.^10,11^ The eMethods in the Supplement includes additional details of sample collection, processing, and assays.

Because we collected blood samples in the patient’s home and shipped the samples to a central laboratory, we conducted a simulation study to compare biomarker levels if the sample was collected, centrifuged, and stored immediately at –80 °C and if the sample was collected using procedures used for a home visit in our study.^12^ We observed less than 6% difference in biomarker levels using these approaches.

### Reference Values for Biomarkers

We used 3 approaches to determine reference values for circulating biomarkers in adults without an infection (eTable 1 in the Supplement). These approaches included using the 95th percentile reported by a clinical laboratory, a previous study,^12^ or the 75th or 95th percentile reported in the literature. The values varied across these approaches, and we used the highest value as reference for our study.

### Statistical Analysis

For each biomarker, we generated box plots of observed levels at the 5 time points and calculated the proportion that exceeded the reference value. Samples were obtained in most patients during hospitalization but could not be obtained from many patients after discharge, resulting in instances in which there were early measures but no subsequent measures (dropout) or there were missing observations between successful collections (eg, measures at 3 and 12 months but none at 6 months). To identify biomarker trajectory groups during the in-hospital course, we used a latent class mixture model.^13^ The joint latent class mixture model (JLCMM) simultaneously models biomarker trajectories and time to dropout, reducing bias induced by the association between biomarker values and occurrence of dropout. Additional details are included in the eMethods in the Supplement.

After identification of trajectories for individual biomarkers, we identified the biomarkers with persistent immune dysregulation as those with mean values of at least 1 trajectory consistently higher than the reference range. Of the 9 biomarkers, only hs-CRP and sPDL1 met this criterion. We then cross-tabulated the classes for these 2 biomarkers to create groups or phenotypes. Because the biomarker data were missing for many patients after hospital discharge, we examined biomarker patterns stratified by phenotype among patients for whom at least 1 biomarker measurement was available after hospital discharge.

Once phenotypes were defined, we modeled their association with long-term outcomes. We chose models based on the frequency of outcomes, appropriateness of distributional assumptions (eg, proportionality of hazards), and need to incorporate competing risk. We used logistic regression to estimate odds ratios (ORs) to compare all-cause mortality, Cox proportional hazards regression models to estimate hazard ratios (HRs) to compare all-cause readmission or mortality, and the Fine-Gray model to estimate subdistribution HRs (SHRs) to compare cause-specific readmission or mortality in the presence of competing risk of death due to other causes. Because the proportional hazards assumption was violated for the Cox and Fine-Gray models, we estimated different HRs and SHRs and their 95% CIs at prespecified intervals (0-6 months and 6 months to 1 year) based on prior studies.^6,14^ All models included the following covariates: age, sex, race/ethnicity, chronic disease, illness severity, organ support, and site of infection.

We conducted sensitivity analyses by age, chronic diseases, cancer, source of infection, time window to ascertain organ failure, and those with at least 1 biomarker during hospitalization and after discharge. We examined the distribution of phenotypes and the magnitude of associations between phenotypes and long-term adverse outcomes.

We also generated cumulative incidence plots for all-cause and cause-specific readmission and mortality stratified by phenotype. We defined 2 covariate patterns and generated plots for these patients by assessing their survival functions using the models developed in the entire sample. We used a bootstrapping procedure to generate CIs. Analyses were performed using the R statistical software, version 3.4.3 (R Project for Statistical Computing) and statistical package lcmm for latent class modeling. Two-sided significance testing was used, and *P* < .05 was considered to be statistically significant.

## Results

Of the 554 patients enrolled, 71 patients were excluded (38 died before hospital discharge, 7 withdrew consent, and data for sepsis admission were incomplete for 26 patients) (eFigure 1 in the Supplement). The remaining 483 patients with sepsis (mean [SD] age, 60.5 [15.2] years; 265 [54.9%] male) who survived to hospital discharge formed the analysis cohort.

### Clinical Characteristics of Survivors of Hospitalization for Sepsis

The clinical characteristics of the patients are given in Table 1. Patients had high burden of chronic diseases, as evidenced by a mean Charlson Comorbidity Index score of 2.2, and 375 (77.6%) had at least 1 chronic disease. A site of infection was identified in 355 patients (73.5%), and the common sites of infection were lung (102 [21.1%]), urinary tract (94 [19.5%]), and abdomen (84 [17.4%]). Of the 483 patients, 298 (61.7%) met criteria for organ dysfunction within 24 hours and 416 (86.1%) within 48 hours. At enrollment, the mean (SD) Acute Physiologic Assessment and Chronic Health Evaluation (APACHE) II score was 12.6 (6.1), and the mean (SD) Sequential Organ Failure Assessment (SOFA) score was 4.2 (3.0). Organ support was common during hospitalization, and 194 (40.2%) patients received vasopressors, 91 (18.8%) received mechanical ventilatory support, and 42 (8.7%) were undergoing dialysis. The mean (SD) length of stay was 10.6 (10.7) days; 428 (88.4%) patients were discharged home, and 56 (11.6%) were discharged to a long-term care facility.

**Table 1.** Clinical Characteristics Before and During Sepsis Admission^a^

Variable	Value (N = 483)
Demographics	
Age, y	
Median (IQR)	61.0 (52.0-71.1)
Mean (SD)	60.5 (15.2)
Male	265 (54.9)
Race/ethnicity	
White	397 (82.2)
Black	65 (13.5)
Other	25 (5.2)
Chronic health conditions	
Charlson Comorbidity Index score	
Median (IQR)	1.0 (0.0-3.0)
Mean (SD)	2.2 (2.4)
Hypertension	278 (57.6)
Myocardial infarction	39 (8.1)
Congestive heart failure	74 (15.5)
Cerebral vascular disease	34 (7.1)
Peripheral vascular disease	40 (8.4)
Chronic kidney disease	102 (21.3)
Chronic dialysis	33 (6.9)
Cirrhosis	25 (5.2)
Chronic pulmonary disease	110 (23.0)
Cancer	82 (17.2)
Medications	
Anticoagulants	105 (21.9)
Statins	167 (34.9)
Steroids	89 (18.6)
Infection site^b^	
Lung (pneumonia)	102 (21.1)
Urinary tract	94 (19.5)
Intra-abdominal	84 (17.4)
Skin and soft tissue	48 (9.9)
Catheter-related	19 (3.9)
Central nervous system	1 (0.2)
Endocarditis	7 (1.4)
Illness severity	
APACHE II score	
Median (IQR)	11.0 (8.0-16.0)
Mean (SD)	12.6 (6.1)
Total SOFA score	
Median (IQR)	4.0 (2.0-6.0)
Mean (SD)	4.2 (3.0)
Cardiovascular SOFA score	
Median (IQR)	1.0 (0.0-1.0)
Mean (SD)	0.8 (0.9)
Central nervous system SOFA score	
Median (IQR)	0.0 (0.0-0.0)
Mean (SD)	0.4 (0.9)
Coagulation SOFA score	
Median (IQR)	0.0 (0.0-1.0)
Mean (SD)	0.6 (0.9)
Liver SOFA score	
Median (IQR)	0.0 (0.0-1.0)
Mean (SD)	0.4 (0.8)
Kidney SOFA score	
Median (IQR)	1.0 (0.0-2.0)
Mean (SD)	1.4 (1.3)
Respiration SOFA score	
Median (IQR)	0.0 (0.0-0.0)
Mean (SD)	0.6 (1.2)
Organ support	
Mechanical ventilation	91 (18.8)
Vasopressor use	194 (40.2)
Dialysis^c^	42 (8.7)
Hospital stay, d	
Median (IQR)	7.0 (5.0-12.0)
Mean (SD)	10.6 (10.7)

Abbreviations: APACHE, Acute Physiologic Assessment and Chronic Health Evaluation; IQR, interquartile range; SOFA, Sequential Organ Failure Assessment.

^a^ Data are presented as number (percentage) of patients unless otherwise indicated.

^b^ Numbers do not total 483 because 128 patients had other sources of infection or the source of infection was not identified.

^c^ Excludes patients who were receiving dialysis before sepsis admission.

Readmissions were common, and a total of 485 readmissions occurred in 205 patients (42.5%) within 1 year. The median time to first readmission was 63 days (interquartile range [IQR], 33.0-129.0 days). Of the patients who were readmitted, 101 (49.3%) were readmitted once, whereas 39 (19.0%) were readmitted twice and 65 (31.7%) were readmitted 3 or more times. Hospital records were available for 447 of the 485 readmissions (92.2%) to ascertain a primary cause, and common causes were infection (223 [49.9%]), cardiovascular disease (43 [9.6%]), and cancer (20 [4.5%]). Other reasons (eTable 2 in the Supplement) accounted for the remaining 161 readmissions (36.0%). The median times to readmission were 74.0 days (IQR, 37.5-160.0 days) for infection, 171.2 days (IQR, 38.8-268.0 days) for cardiovascular disease, and 93.0 days (IQR, 54.5-244.0 days) for cancer.

Of the 483 patients, 43 (8.9%) died by 3 months, 56 (11.6%) died by 6 months, and 85 (17.6%) died by 12 months. The cause of death was ascertained in 69 of 85 deaths (81.2%), and common causes included cancer (32 [46.4%]), cardiovascular disease (12 [17.4%]), and infection (16 [23.2%]). Other causes (eTable 3 in the Supplement) accounted for the remaining 13% of deaths.

### Circulating Biomarker Levels Over Time

Of the 483 patients, 311 (64.4%) were eligible for a home visit at 3 months, 302 (62.5%) at 6 months, and 301 (62.3%) at 12 months because patients died, were readmitted, withdrew, or became residents of long-term care facilities (eFigure 1 in the Supplement). A blood sample was obtained from 110 patients (35.4%) eligible for a home visit at 3 months, 97 (32.1%) eligible for a home visit at 6 months, and 98 (32.6%) eligible for a home visit at 12 months.

A total of 165 patients (34.2%) had at least 1 postdischarge sample, 98 (20.3%) had at least 2, and 42 (8.7%) had at least 3. Twenty-five patients (5.1%) had samples at all 5 time points. The clinical characteristics of patients who did and did not have a sample after hospital discharge are given in eTable 4 in the Supplement. In general, these 2 groups had similar demographics and chronic diseases. However, those with a biomarker measurement after discharge were more likely to have intra-abdominal infections and more likely to receive vasopressors compared with those without a biomarker measurement after discharge.

**Figure 1.**
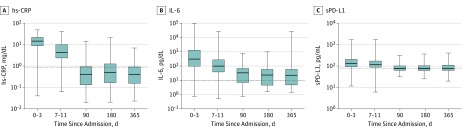
Inflammatory and Immunosuppression Biomarker Values Collected at Each Scheduled Collection Time Point Horizontal dotted lines represent the estimated 95th percentile of biomarker distribution among individuals without an infection. The lowest whisker indicates the minimum value; bottom border of the box, 25th percentile; line bisecting the shaded region of each box, median; top border of the box, 75th percentile; and highest whisker, maximum value. hs-CRP indicates highly sensitive C-reactive protein; IL, interleukin; and sPD-L1, soluble programmed death ligand 1.

Circulating inflammatory biomarkers were elevated at 3, 6, and 12 months in a large proportion of sepsis survivors (Figure 1 and eTable 5 in the Supplement). A total of 72 patients (74.2%) at 3 months, 62 (70.5%) at 6 months, and 59 (66.3%) at 12 months had elevated IL-6 levels. For hs-CRP, 23 (25.8%) had elevated levels at 3 months, 26 (30.2%) at 6 months, and 23 (25.6%) at 12 months. For sPD-L1, 45 (46.4%) had elevated levels at 3 months, 40 (44.9%) at 6 months, and 44 (49.4%) at 12 months. Levels of other circulating biomarkers are shown in eFigure 2 in the Supplement. The total number of patients who had at least 1 elevated biomarker sample after hospital discharge were 72 (46.8%) for E-selectin, 35 (25.0%) for intercellular adhesion molecule 1, 34 (23.0%) for vascular cell adhesion molecule 1, 56 (36.4%) for plasminogen activator inhibitor 1, 57 (40.0%) for D-dimer, and 54 (32.7%) for nitrate.

### Biomarker Trajectories

**Figure 2.**
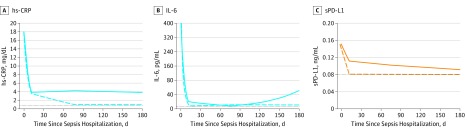
Latent Trajectory Classes for Biomarkers of Inflammation and Immunosuppression Estimated During 1 Year Using Joint Latent Class Mixture Models (JLCMM) Horizontal dotted lines represent the estimated 95th percentile of biomarker distribution among individuals without an infection. The solid and dashed lines indicate latent mean longitudinal biomarker trajectories corresponding to the 2 classes identified by separate joint latent class mixture models fit to each set of biomarker data. hs-CRP indicates highly sensitive C-reactive protein; IL, interleukin; and sPD-L1, soluble programmed death ligand 1.

Most patients had a single latent trajectory for the 9 biomarkers during the in-hospital course (eFigure 3 in the Supplement) using JLCMMs. In contrast, we identified 2 classes for each biomarker based on their latent trajectories during the first 6 months using the JLCMM (Figure 2 and eFigure 4 in the Supplement). For example, we observed trajectories that had consistently high or normal levels of hs-CRP (334 of 477 [70.0%] had high hs-CRP levels and 143 of 477 [30.0%] had normal hs-CRP levels) and had high or normal levels of sPDL1 (330 of 479 [68.9%] had high sPDL1 levels and 149 of 479 [31.1%] had normal sPDL1 levels). The mean (SD) posterior probabilities of membership to the high and normal hs-CRP trajectories were 96.3% (5.8%) and 98.4% (5.3%), and the mean (SD) posterior probabilities of membership to the high and normal sPDL1 trajectories were 95.5% (6.6%) and 97.7% (7.7%). We observed similar latent trajectories for hs-CRP and sPDL1 in subgroup analysis restricted to 337 (69%) patients who had clinical follow-up (patients died or were readmitted) or a biomarker measurement after hospital discharge (eFigure 5 in the Supplement). In this subset, we observed latent trajectories that had consistently high levels of hs-CRP in 190 patients (56.9%) and sPDL1 in 215 patients (64.4%).

We identified 2 latent trajectories for IL-6, but levels were variable during 6 months. For the remaining biomarkers, either both trajectories had levels below the reference range for most patients during the 6 months (eg, intercellular adhesion molecule 1, vascular cell adhesion molecule 1, plasminogen activator inhibitor 1, and nitrate) or a single trajectory had levels above the reference range only briefly (eg, E-selectin and D-dimer).

### Phenotypes

On the basis of trajectories for hs-CRP and sPDL1, we identified 4 phenotypes: patients with high hs-CRP and sPDL1 levels (326 of 477 [68.3%]) and referred to as hyperinflammation or immunosuppression phenotype, those with normal hs-CRP and sPDL1 (141 of 477 [29.6%]) and referred to as normal phenotype, a small number who had hyperinflammation only (8 of 477 [0.02%]), and a small number who had immunosuppression only (2 of 477 [0.004%]). The distribution of biomarker levels for hs-CRP and sPDL1 for patients who had at least 1 biomarker measurement after hospital discharge and stratified by hyperinflammation and immunosuppression and normal phenotypes is shown in eFigure 6 and eFigure 7 in the Supplement.

We observed similar distribution of the hyperinflammation and immunosuppression, normal, hyperinflammation only, and immunosuppression only phenotypes when results were stratified by age, chronic diseases, cancer, source of infection, and time window to ascertain organ failure (eTable 6 in the Supplement). Because only a small proportion of patients belonged to the hyperinflammation only and immunosuppression only phenotypes, subsequent analyses excluded them.

Patients with the hyperinflammation and immunosuppression phenotype had similar clinical characteristics and in-hospital course compared with the patients with the normal phenotype (Table 2). The age and chronic disease burden were similar (mean [SD] age, 59.7 [15.7] years in the hyperinflammation and immunosuppression phenotype group and 61.5 [14.0] years in the normal phenotype group; the median Charlson Comorbidity Index score was 1.0 [IQR, 0.0-3.0] for both groups). The illness severity was also similar, as evidenced by similar median APACHE II scores (11.5 [IQR, 8.0-17.0] in the hyperinflammation and immunosuppression phenotype group and 11.0 [IQR, 8.0-15.0] in the normal phenotype group) and total SOFA scores (4.0 [IQR, 2.0-6.0] in the hyperinflammation and immunosuppression phenotype group and 3.0 [IQR, 2.0-6.0] in the normal phenotype group). Those with the hyperinflammation and immunosuppression phenotype were less likely to have an intra-abdominal infection (46 [14.1%] vs 36 [25.5%]) and more likely to have skin and soft-tissue infections (40 [12.3%] vs 7 [5.0%]), whereas the frequency of lung infections was similar (64 [19.6%] in the hyperinflammation and immunosuppression phenotype group and 35 [24.8%] in the normal phenotype group). Small differences were seen in the frequency of organ support (63 patients [19.3%] in the hyperinflammation and immunosuppression phenotype group and 22 [15.6%] in the normal phenotype group received mechanical ventilatory support) and length of stay (mean [SD], 11.2 [10.8] days in the hyperinflammation and immunosuppression phenotype group and 8.8 [9.1] days in the normal phenotype group).

**Table 2.** Comparison of Clinical Characteristics Between 2 Phenotypes Based on Inflammation and Immunosuppression Biomarker Trajectories During 6 Months^a^

Variable	Hyperinflammation and Immunosuppression Phenotype (n = 326)	Normal Phenotype (n = 141)	*P* Value^b^
Demographics			
Age, y			
Median (IQR)	60.7 (51.3-70.9)	61.4 (54.3-71.2)	.36
Mean (SD)	59.7 (15.7)	61.5 (14.0)	
Female sex	142 (43.6)	68 (48.2)	.41
Race/ethnicity			
White	267 (81.9)	119 (84.4)	.60
Black	43 (13.2)	18 (12.8)	>.99
Other	18 (5.5)	4 (2.8)	.24
Chronic health conditions			
Charlson Comorbidity Index score			
Median (IQR)	1.0 (0.0-3.0)	1.0 (0.0-3.0)	.31
Mean (SD)	2.3 (2.5)	1.9 (2.2)	
Hypertension	191 (59.1)	78 (55.7)	.56
Myocardial infarction	25 (7.7)	12 (8.6)	.91
Congestive heart failure	53 (16.4)	20 (14.4)	.68
Cerebral vascular disease	24 (7.4)	9 (6.4)	.85
Peripheral vascular disease	30 (9.3)	10 (7.1)	.57
Chronic kidney disease	70 (21.7)	27 (19.4)	.67
Chronic dialysis	23 (7.1)	8 (5.8)	.73
Cirrhosis	20 (6.2)	4 (2.9)	.17
Chronic pulmonary disease	70 (21.7)	37 (26.4)	.32
Cancer	57 (17.4)	24 (17)	.23
Medications			
Anticoagulants	75 (23.2)	28 (19.9)	.50
Statins	111 (34.5)	51 (36.2)	.81
Steroids	56 (17.4)	28 (19.9)	.62
Infection site^c^			
Lung (pneumonia)	64 (19.6)	35 (24.8)	.26
Urinary tract	62 (19)	26 (18.4)	.99
Abdomen	46 (14.1)	36 (25.5)	<.01
Skin and soft tissue	40 (12.3)	7 (5.0)	.02
Catheter related	13 (4)	5 (3.5)	>.99
Central nervous system	1 (0.3)	0	>.99
Endocarditis	7 (2.1)	0	.11
Illness severity			
APACHE II score			
Median (IQR)	11.5 (8.0-17.0)	11.0 (8.0-15.0)	.18
Mean (SD)	12.8 (6.1)	11.9 (5.6)	
Total SOFA score			
Median	4.0 (2.0-6.0))	3.0 (2.0-6.0)	.39
Mean (SD)	4.3 (3)	4 (2.8)	
Cardiovascular SOFA score			
Median (IQR)	1.0 (0.0-1.0)	1.0 (0.0-1.0)	.50
Mean (SD)	0.7 (0.8)	0.8 (0.9)	
Central nervous system SOFA score			
Median (IQR)	0 (0.0-0.0)	0 (0.0-0.0)	.40
Mean (SD)	0.4 (0.9)	0.3 (0.8)	
Coagulation SOFA score			
Median (IQR)	0 (0.0-0.0)	0 (0.0-0.0)	.80
Mean (SD)	0.6 (1)	0.6 (0.8)	
Liver SOFA score			
Median (IQR)	0 (0.0-1.0)	0 (0.0-1.0)	.76
Mean (SD)	0.4 (0.9)	0.4 (0.8)	
Renal SOFA score			
Median (IQR)	1.0 (0.0-2.0)	1.0 (0.0-2.0)	.23
Mean (SD)	1.4 (1.3)	1.2 (1.3)	
Respiration SOFA score			
Median (IQR)	0 (0.0-0.0)	0 (0.0-0.0)	.86
Mean (SD)	0.6 (1.2)	0.6 (1.2)	
Organ support			
Mechanical ventilatory support	63 (19.3)	22 (15.6)	.41
Vasopressor use	126 (38.7)	62 (44)	.33
Dialysis^d^	32 (9.8)	7 (5)	.12
Hospital stay, d			
Median (IQR)	8.0 (5.0-14.0)	6.0 (4.0-10.0)	.01
Mean (SD)	11.2 (10.8)	8.8 (9.1)	

Abbreviations: APACHE, Acute Physiologic Assessment and Chronic Health Evaluation; IQR, interquartile range; SOFA, Sequential Organ Failure Assessment.

^a^ Data are presented as number (percentage) of patients unless otherwise indicated.

^b^ *P* values generated from χ^2^ tests for categorical variables, and Wilcoxon tests for continuous variables.

^c^ Numbers do not add to 483 because 128 patients had other sources of infection or the source of infection was not identified.

^d^ Excludes patients who received dialysis before sepsis admission.

### Association Between Phenotypes and Outcomes

Compared with the normal phenotype, those with the hyperinflammation and immunosuppression phenotype had higher 1-year mortality (adjusted OR, 8.26; 95% CI, 3.45-21.69; *P* < .001) (Table 3). The Kaplan-Meier curves for these phenotypes are shown in eFigure 8 in the Supplement. Those with the hyperinflammation and immunosuppression phenotype had a higher risk of all-cause readmission or mortality, but the higher risk persisted only during the initial 6 months (adjusted HR, 1.53; 95% CI, 1.10-2.13; *P* = .01). Those with the hyperinflammation and immunosuppression phenotype had a higher risk of readmission or mortality due to cardiovascular disease (SHR, 5.07; 95% CI, 1.18-21.84; *P* = .02) and cancer (SHR, 5.15; 95% CI, 1.25-21.18; *P* = .02) during 6 months but not readmission or mortality due to infection. The magnitude of most associations was unchanged in sensitivity analyses restricted to patients for whom the probability of phenotype membership was 80% or higher, those with organ dysfunction within 24 hours of admission, and in subgroups based on age, chronic diseases, and infection source (eTables 7-9 in the Supplement). The reasons for readmission and death stratified by phenotype are given in eTable 10 and eTable 11 in the Supplement.

**Table 3.** Association Between the Hyperinflammation and Immunosuppression and Normal Phenotypes and Long-term Outcomes

Variables	No. of Events/No. at Risk (%)		Adjusted OR, HR, or SHR (95% CI)^a^	*P* Value
	Hyperinflammation and Immunosuppression Phenotype	Normal Phenotype		
All-cause 1-y mortality	77/326 (23.4)	6/141 (4.3)	8.26 (3.45-21.69)	<.001
All-cause readmission or death, d				
0-180	144/326 (44.2)	48/141 (34.0)	1.53 (1.10-2.13)	.01
181-365	30/182 (16.5)	15/93 (16.1)	1.18 (0.64-2.20)	.59
Readmission or death due to infection, d				
0-180	79/326 (24.2)	27/141 (19.1)	1.35 (0.87-2.10)	.18
181-365	22/217 (10.1)	5/114 (4.5)	2.00 (0.75-5.36)	.17
Readmission or death due to cardiovascular disease, d				
0-180	22/326 (6.7)	2/141 (1.4)	5.07 (1.18-21.84)	.02
180-365	6/258 (2.3)	7/139 (5.0)	0.42 (0.14-1.28)	.13
Readmission or death due to cancer, d				
0-180	25/326 (7.7)	2/141 (1.4)	5.15 (1.25-21.18)	.02
180-365	7/269 (2.6)	5/139 (3.6)	0.67 (0.20-2.27)	.53

Abbreviations: HR, hazard ratio; OR, odds ratio; SHR, subdistribution hazard ratio.

^a^ The ORs were estimated using logistic regression model for all-cause 1-year mortality. The HRs were estimated using a Cox proportional hazards regression model for all-cause 1-year readmission or death. The SHRs were estimated using the Fine-Gray model for cause-specific analyses of death or readmission. Covariates included age, sex, race/ethnicity, Charlson Comorbidity Index score, APACHE II score, infection site, mechanical ventilatory support, vasopressor use, and dialysis.

Cumulative incidence of all-cause and cause-specific readmission and mortality stratified by phenotype for 2 hypothetical patient scenarios is shown in eFigure 9 in the Supplement. In each scenario, readmissions or mortality for any reason, cardiovascular disease, or cancer were more frequent in patients with the hyperinflammation and immunosuppression phenotype compared with normal phenotype.

## Discussion

Our results suggest that the dysregulated host immune response activated during sepsis may persist up to 1 year. We identified 2 common phenotypes of sepsis survivors based on circulating hs-CRP and sPDL1 levels; approximately two-thirds had persistent elevation of both inflammation and immunosuppression biomarkers, and a third had normal biomarkers. These groups had similar clinical characteristics before the index hospitalization for sepsis and in-hospital course. However, those with persistent hyperinflammation and immunosuppression had a higher risk of readmission and mortality, particularly due to cardiovascular disease and cancer. These results were robust in multivariable analyses adjusting for potential confounders and several sensitivity analyses.

Our findings have important implications. First, the persistence of hyperinflammation in a large number of sepsis survivors and the increased risk of cardiovascular events among these patients may explain the association between infection and cardiovascular disease in a prior study.^15^ Second, our findings have important implications for designing future sepsis trials. Although prior trials tested immunomodulation strategies during only the early phase of hospitalization for sepsis, immunomodulation may be needed after hospital discharge. Third, our findings suggest potential mechanisms to explore in future studies to understand long-term sequelae in sepsis survivors. A prior study^16^ performed during hospitalization for sepsis found that a phenotype similar to the hyperinflammation and immunosuppression phenotype was common during hospitalization for sepsis and associated with 90-day mortality. In contrast to that previous study,^16^ we did not measure IL-10 as a marker of immunosuppression because IL-10 levels were below the detection threshold of the assay in a large number of patients. Instead, we measured sPDL1 levels because recent work^17,18^ suggests that it is important and may play a role in chronic critical illness. Future studies should examine the role of other biomarkers within the PD1 pathway, including PD1 receptor expression on immune cells and circulating PD1 levels. Although we chose biomarkers in 5 pathways, only 2 biomarkers within inflammation and immunosuppression pathways identified common phenotypes associated with adverse events. Potential reasons include possibly underpowering for the detection of the role of biomarkers or the potential role of other biomarkers within these 3 additional pathways.

We considered several confounders that may explain persistently high biomarker levels in our study. First, patients may have had high hs-CRP and sPDL1 levels before sepsis occurred rather than a consequence of sepsis. It is not practical to measure these biomarkers before and immediately after sepsis in a large cohort. However, a previous study^8^ used a murine cecal ligation and puncture model in which mice were randomly allocated to cecal ligation and puncture or sham surgery. The study^8^ found that circulating inflammatory markers were 4-fold higher at 5 months in mice who underwent cecal ligation and puncture compared with sham surgery. In humans, higher levels of chronic inflammation have been attributed to aging (inflammaging) or chronic diseases.^19^ However, the mean age and chronic disease burden was similar among patients with the hyperinflammation and immunosuppression phenotype compared with those with the normal phenotype. In addition, the frequency of patients with the hyperinflammation and immunosuppression phenotype was high even among younger individuals and those without a chronic disease. Thus, the higher levels observed after hospital discharge in our study cannot be solely attributable to high circulating levels before sepsis. Second, we considered that higher levels may be attributable to readmissions or additional infections after the index sepsis admission. We excluded follow-up if sepsis survivors resided in long-term care facilities, where subclinical infections are common, and collected biomarker samples at home, ensuring that patients did not have clinical symptoms of infection and were not recently readmitted. Third, biomarker levels may be spuriously elevated because of sample collection or transport. We standardized sample collection methods, ensured that samples were centrifuged immediately after sample collection, and did not observe variation in biomarkers when we conducted stability studies to mimic shipping conditions.^12^

### Strengths and Limitations

The strengths of our study include its multicenter design, use of standardized procedures for sample collection and processing, and multiple approaches that have been used in prior federally funded studies to ascertain mortality and readmission.^9^ Our study also has several limitations. First, many patients had missing data for several time points. Despite using different approaches to ensure follow-up, including obtaining contact information for multiple individuals, contacting patients in the evening and on weekends, and offering monetary compensation, we obtained a blood sample in a small subset of patients. However, we did not see large differences in the prehospitalization and in-hospital clinical characteristics between patients with and without missing biomarkers. We used the JLCMM to account for missing data and identify latent trajectories. This model relied on the assumption that some data were missing at random before dropout whereas other data were missing after dropout and were likely not missing at random. The JLCMM allowed us to simultaneously model time to dropout to account for missingness and to identify latent trajectories. Most missing data were attributable to dropout; thus, our reliance on the missing-at-random assumption was minimal. Instead, our results primarily rely on the correctness of the assumption that the dropout and longitudinal biomarker processes were interdependent. Second, we did not include controls without sepsis in our study because we were interested in the variation in long-term biomarker trajectories within a population with sepsis rather than a comparison of patients with sepsis with patients with other conditions. Nevertheless, the lack of controls without sepsis limits our ability to speculate on the degree to which these trajectories may be seen in other conditions. Third, although we found 2 latent trajectories for most biomarkers, it is possible that patients within each trajectory may be further subdivided based on unmeasured features. Fourth, we did not include data regarding hospitalizations before sepsis. Fifth, we limited collection of clinical data after hospital discharge to mortality and hospital readmissions, and patients may have been diagnosed with chronic diseases in the outpatient settings.

## Conclusions

The dysregulated host immune response activated during sepsis may persist up to 1 year. Individuals with persistent biomarkers of inflammation and immunosuppression had a higher risk of readmission and death due to cardiovascular disease and cancer compared with those with normal circulating biomarkers. Our findings suggest that long-term immunomodulation strategies should be explored in patients hospitalized with sepsis.

## Notes

Supplement. **eMethods.** Supplementary Methods **eFigure 1.** Flowchart of the Study **eFigure 2.** Boxplots of Values for Markers of Endothelial Dysfunction, Hemostasis, and Oxidative Stress **eFigure 3.** Latent Longitudinal Trajectories From Non–Joint Latent-Class Mixture Models (LCMM) Using Biomarker Data From the First 13 Days of the In-hospital Period Only **eFigure 4.** Latent Trajectory Classes for Biomarkers of Hemostasis, Endothelial Dysfunction, and Oxidative Stress **eFigure 5.** JLCMM Model Results Using the 337 Patients With at Least 1 Postdischarge Biomarker Sample or Who Had Died or Were Readmitted Within 1 Year **eFigure 6.** Distribution of hs-CRP and PD-L1 for 4 Periods, Stratified by Phenotypes Identified in the Primary JLCMM Analysis **eFigure 7.** Plots of hs-CRP and PD-L1 Levels Over Time, Among Patients With at Least 1 In-hospital and 1 Postdischarge Sample **eFigure 8.** Kaplan-Meier Plot of Mortality by Phenotype **eFigure 9.** Cumulative Incidence of Readmission and Mortality Stratified by Phenotype for 2 Hypothetical Patient Scenarios **eTable 1.** Rationale for Selection of Reference Value for Individuals Without an Infection **eTable 2.** Reason for Readmission Among Patients in the “Other Diagnoses” Category **eTable 3.** Reason for Death Among Patients in the “Other Diagnoses” Category **eTable 4.** Comparison of Clinical Characteristics Between Subjects With and Without Postdischarge Biomarker Measurement **eTable 5.** Observed Distribution of Circulating Biomarkers by Time Point **eTable 6.** Distribution of Combined Phenotypes Among Patients in 6 Different Subgroups **eTable 7.** Association Between Phenotypes and Long-term Outcomes Among Individuals With Posterior Probability of Membership Into a Phenotype Greater Than 80% in the Primary Analysis **eTable 8.** Association Between Phenotypes and Long-term Outcomes Among Individuals in the Sensitivity Analysis Among Subjects Who Had at Least 1 Postdicharge Biomarker Sample or Who Died or Were Readmitted Within 1 Year (N = 337) **eTable 9.** Association Between Phenotypes as Determined by the Primary Analysis Joint Models and Long-term Outcomes Among 5 Subsets of Individuals With Specific Characteristics **eTable 10.** Reasons for Readmission by Phenotype **eTable 11.** Reasons for Death Stratified by Phenotype **eReferences**
