# Supplementary material for: Long-term Host Immune Response Trajectories Among Hospitalized Patients With Sepsis
Source: JAMA Netw Open. 2019 Aug 7;2(8):e198686. doi: 10.1001/jamanetworkopen.2019.8686 (PMC6686981; doi:10.1001/jamanetworkopen.2019.8686)
Supplement: Supplement. — eMethods. Supplementary Methods eFigure 1. Flowchart of the Study eFigure 2. Boxplots of Values for Markers of Endothelial Dysfunction, Hemostasis, and Oxidative Stress eFigure 3. Latent Longitudinal Trajectories From Non–Joint Latent-Class Mixture Models (LCMM) Using Biomarker Data From the First 13 Days of the In-hospital Period Only eFigure 4. Latent Trajectory Classes for Biomarkers of Hemostasis, Endothelial Dysfunction, and Oxidative Stress eFigure 5. JLCMM Model Results Using the 337 Patients With at Least 1 Postdischarge Biomarker Sample or Who Had Died or Were Readmitted Within 1 Year eFigure 6. Distribution of hs-CRP and PD-L1 for 4 Periods, Stratified by Phenotypes Identified in the Primary JLCMM Analysis eFigure 7. Plots of hs-CRP and PD-L1 Levels Over Time, Among Patients With at Least 1 In-hospital and 1 Postdischarge Sample eFigure 8. Kaplan-Meier Plot of Mortality by Phenotype eFigure 9. Cumulative Incidence of Readmission and Mortality Stratified by Phenotype for 2 Hypothetical Patient Scenarios eTable 1. Rationale for Selection of Reference Value for Individuals Without an Infection eTable 2. Reason for Readmission Among Patients in the “Other Diagnoses” Category eTable 3. Reason for Death Among Patients in the “Other Diagnoses” Category eTable 4. Comparison of Clinical Characteristics Between Subjects With and Without Postdischarge Biomarker Measurement eTable 5. Observed Distribution of Circulating Biomarkers by Time Point eTable 6. Distribution of Combined Phenotypes Among Patients in 6 Different Subgroups eTable 7. Association Between Phenotypes and Long-term Outcomes Among Individuals With Posterior Probability of Membership Into a Phenotype Greater Than 80% in the Primary Analysis eTable 8. Association Between Phenotypes and Long-term Outcomes Among Individuals in the Sensitivity Analysis Among Subjects Who Had at Least 1 Postdicharge Biomarker Sample or Who Died or Were Readmitted Within 1 Year (N = 337) eTable 9. Association Between Phenoty [file jamanetwopen-2-e198686-s001.pdf]

Supplementary Online Content

Yende S, Kellum JA, Talisa VB, et al. Long-term host immune response trajectories among hospitalized patients with sepsis. *JAMA Netw Open*. 2019;2(8):e198686. doi:10.1001/jamanetworkopen.2019.8686

eMethods. Supplementary Methods
eFigure 1. Flowchart of the Study
eFigure 2. Boxplots of Values for Markers of Endothelial Dysfunction, Hemostasis, and Oxidative Stress
eFigure 3. Latent Longitudinal Trajectories From Non–Joint Latent-Class Mixture Models (LCMM) Using Biomarker Data From the First 13 Days of the In-hospital Period Only
eFigure 4. Latent Trajectory Classes for Biomarkers of Hemostasis, Endothelial Dysfunction, and Oxidative Stress
eFigure 5. JLCMM Model Results Using the 337 Patients With at Least 1 Postdischarge Biomarker Sample or Who Had Died or Were Readmitted Within 1 Year
eFigure 6. Distribution of hs-CRP and PD-L1 for 4 Periods, Stratified by Phenotypes Identified in the Primary JLCMM Analysis
eFigure 7. Plots of hs-CRP and PD-L1 Levels Over Time, Among Patients With at Least 1 In-hospital and 1 Postdischarge Sample
eFigure 8. Kaplan-Meier Plot of Mortality by Phenotype
eFigure 9. Cumulative Incidence of Readmission and Mortality Stratified by Phenotype for 2 Hypothetical Patient Scenarios
eTable 1. Rationale for Selection of Reference Value for Individuals Without an Infection
eTable 2. Reason for Readmission Among Patients in the "Other Diagnoses" Category
eTable 3. Reason for Death Among Patients in the "Other Diagnoses" Category
eTable 4. Comparison of Clinical Characteristics Between Subjects With and Without Postdischarge Biomarker Measurement
eTable 5. Observed Distribution of Circulating Biomarkers by Time Point
eTable 6. Distribution of Combined Phenotypes Among Patients in 6 Different Subgroups
eTable 7. Association Between Phenotypes and Long-term Outcomes Among Individuals With Posterior Probability of Membership Into a Phenotype Greater Than 80% in the Primary Analysis
eTable 8. Association Between Phenotypes and Long-term Outcomes Among Individuals in the Sensitivity Analysis Among Subjects Who Had at Least 1 Postdicharge Biomarker Sample or Who Died or Were Readmitted Within 1 Year (N=337)
eTable 9. Association Between Phenotypes as Determined by the Primary Analysis Joint Models and Long-term Outcomes Among 5 Subsets of Individuals With Specific Characteristics
eTable 10. Reasons for Readmission by Phenotype
eTable 11. Reasons for Death Stratified by Phenotype
eReferences

This supplementary material has been provided by the authors to give readers additional information about their work.

© 2019 Yende S et al. *JAMA Network Open.*

eMethods. Supplementary Methods

A. Design and sites

We conducted a prospective, observational cohort study of patients hospitalized with sepsis from 2012 to 2017. All participating sites were in the United States and are listed below:

1. University of Pittsburgh Medical Center – Presbyterian and Montefiore hospitals, Pittsburgh, Pennsylvania
2. Allegheny General hospital, Pittsburgh, Pennsylvania
3. Veterans Affairs Pittsburgh Healthcare System, Pittsburgh, Pennsylvania
4. Massachusets General hospital, Boston, Massachusets
5. Beth Israel Deaconess Medical Center, Boston, Massachusets
6. Brigham and Women's hospital, Boston, Massachusets
7. George Washington University hospital, Washington, District of Columbia
8. Maricopa Medical Center, Tuscon, Arizona
9. Ohio State University – Wexner Medical Center, Columbus, Ohio
10. Intermountain Medical Center, Salt Lake, Utah
11. University of Alabama hospital, Birmingham, Alabama
12. Norwalk hospital, Norwalk, Connecticut

© 2019 Yende S et al. *JAMA Network Open.*

B. Eligibility criteria

Subjects were included if they had a suspected infection and met at least 1 of the following criteria for organ dysfunction:

1. Cardioascular: systolic blood pressure was < 90 mmHg, mean arterial pressure was ≤ 70mm Hg, the lactate level is >4 mMol/L, or subject received vasopressors
2. Renal: Increase in serum creatinine by >0.3 mg/dl (>26.5 umol/l)within 48 hours, increase in serum creatinine >1.5 times baseline, which is known or presumed to be normal, or urine volume <0.5 ml/kg/hour for 6 hours.
3. Coagulation: platelet count was <80,000/mm3 or decreased by 50% in the 3 preceding days
4. Liver: bilirubin > 2.0 mg/dl.
5. Respiratory: PaO2/FiO2 = < 250 or SpO2/FiO2= <274
6. Central nervous system: Glasgow comma score was < 12.

Subjects were excluded if they have any of the following criteria:

1. Known pregnancy
2. CD4 < 50/mm^3
3. Treating physician deems aggressive care unsuitable
4. The primary reasons for hospitalization were acute cerebral vascular event or acute coronary syndrome.
5. Subject or next of kin (who resides with the subject) do not comprehend English

© 2019 Yende S et al. *JAMA Network Open.*

6. Unable to conduct follow-up home visits because subject resides in a long-term care facility (e.g. nursing home or skilled nursing facility), was homeless, or lacked reliable contact information to schedule follow-up visits

C. Readmissions

We asked patients about all overnight hospital planned and unplanned admissions at 3, 6, and 12 months. Once an admission was reported, we obtained medical records, including a history and physical examination, discharge summary, and relevant radiologic and laboratory findings. Additionally, we queried the health records within the healthcare systems where the patients were enrolled. We centrally adjudicated a primary and secondary cause for each readmission using a structured approach.[1] Two reviewers determined the cause of readmission for a subset (n=126), and Cohen's kappa showed good agreement between raters (K=0.79, p<0.0001). All cause-specific readmission analyses included only unplanned readmissions.

D. Long-term mortality

We determined if patients had died using telephone follow-up, review of hospitalization records, and National Death Index search. Death was confirmed by obtaining death certificates whenever possible. We adjudicated cause of death by review of hospital records, next-of-kin interviews, and National Death Index records.

E. Laboratory procedures

We assessed the host immune response by measuring biomarkers at 5 time points during the index hospitalization (0-72 hours and 7-11 days) and at home (3, 6, and 12 months). The study coordinators at each site collected in-hospital samples and home health coordinators collected samples during home visits. After sample collection, the samples were centrifuged in the

laboratory, aliquoted, stored at -4°C, and shipped in dry ice from each site, and were centrifuged immediately using portable centrifuges and shipped overnight using cold packs (~4°C) from the patient's home. The samples were stored at -80°C at the University of Pittsburgh until biomarker analyses were performed.

We used the Bio-Plex 200 instrument (Bio-Rad Laboratories, Hercules, CA) and multiplex assays to measure circulating concentrations of plasma IL-6 and PAI-1 (Bio-Rad Laboratories, Hercules, CA) and E-selectin, sICAM-1, and sVCAM-1 (R&D Systems, Minneapolis, MN). We used the nitrate/nitrite colorimetric kit (Cayman Chemical, Ann Arbor, MI) to measure plasma nitrate concentrations spectrophotometrically (Synergy™ Mx MultiMode Microplate Reader, BioTek Instruments, Inc., Vermont). We measured sPDL-1 using ELISA (R&D Systems, Minneapolis, MN). We used the AU5800 automated analyzer (Beckman Coulter, Brea, CA) to measure concentrations of hs-CRP and the STA-R® Evolution analyzer (Diagnostica Stago, Inc., Parsippany, NJ) to measure D-dimer. Assays were performed by the University of Pittsburgh research or clinical laboratory.

F. Statistical analysis

We calculated descriptive statistics for clinical characteristics during the index sepsis admission and for readmissions and mortality up to 1 year. For each biomarker, we generated box plots of observed levels at the 5 time points and calculated the proportion that exceeded the reference value. Samples could not be obtained in many patients after hospital discharge, resulting in instances where there were early measures but none subsequently (dropout) or instances where there were missing observations between successful collections (e.g., measures at 3 and 12 months, but none at 6 months).

© 2019 Yende S et al. *JAMA Network Open.*

In order to identify biomarker trajectory groups in the presence of missing data, we used joint latent-class mixture models (JLCMM). The JLCMM simultaneously models biomarker trajectories and time to dropout, eliminating bias induced by the correlation between biomarker values and occurrence of dropout. It also handles intermittent missing observations through the inclusion of random effects and the use of empirical Bayes estimates.[2] Because the biomarker values were not normally distributed, we transformed the data using either log or spline functions. We modeled dropout using a Weibull model with group-specific baseline hazards, and included the following covariates: age, infection site, dialysis, vasopressor use, mechanical ventilation, acute physiology and chronic health evaluation (APACHE) II score, and total sequential organ failure assessment (SOFA) score because these factors may explain time to dropout. We did not include covariates in the longitudinal model of biomarkers to limit the influence of clinical characteristics on biomarker trajectory classes. We determined the optimal number of latent classes (trajectories) using the Bayesian information criterion (BIC).

Following identification of trajectories for individual biomarkers, we selected biomarkers with persistent immune dysregulation if the mean values of at least one trajectory for a biomarker were consistently higher than the reference range. Since more than one biomarker met this criterion, we explored patterns of trajectories across biomarkers. We determined the predicted probability of membership in each pattern by estimating the product of the probabilities of individual biomarker trajectory. These patterns were referred to as phenotypes and each patient was assigned to a phenotype.

We compared the frequency of these phenotypes in the overall sample and stratified by age and by presence of chronic diseases to ensure that the distribution of the phenotypes was similar across different age groups and in those with and without chronic diseases. We also compared

© 2019 Yende S et al. *JAMA Network Open.*

the clinical characteristics of these phenotypes to determine whether baseline or hospital course affected phenotype assignment.

We chose models to determine the association between phenotypes and outcomes based on frequency of outcomes, proportionality assumptions being met, and need to incorporate competing risk. We used logistic regression to estimate odds ratios (ORs) to compare all-cause mortality, Cox models to estimate hazard ratios (HRs) to compare all-cause readmission or mortality, and the Fine-Gray model to estimate subdistribution HRs to compare cause-specific readmission or mortality in the presence of competing risk of death due to other causes. Because the proportional hazards assumption was violated for the Cox and Fine-Gray models, we estimated different hazard ratios (HRs) and their 95% CIs at prespecified intervals (0-6 months and 6 months-1 year) based on prior studies.[3,4] All models included the following covariates: age, sex, race, chronic disease, illness severity, organ support, and site of infection.

© 2019 Yende S et al. *JAMA Network Open.*

II. FIGURES

eFigure 1. Flowchart of the study

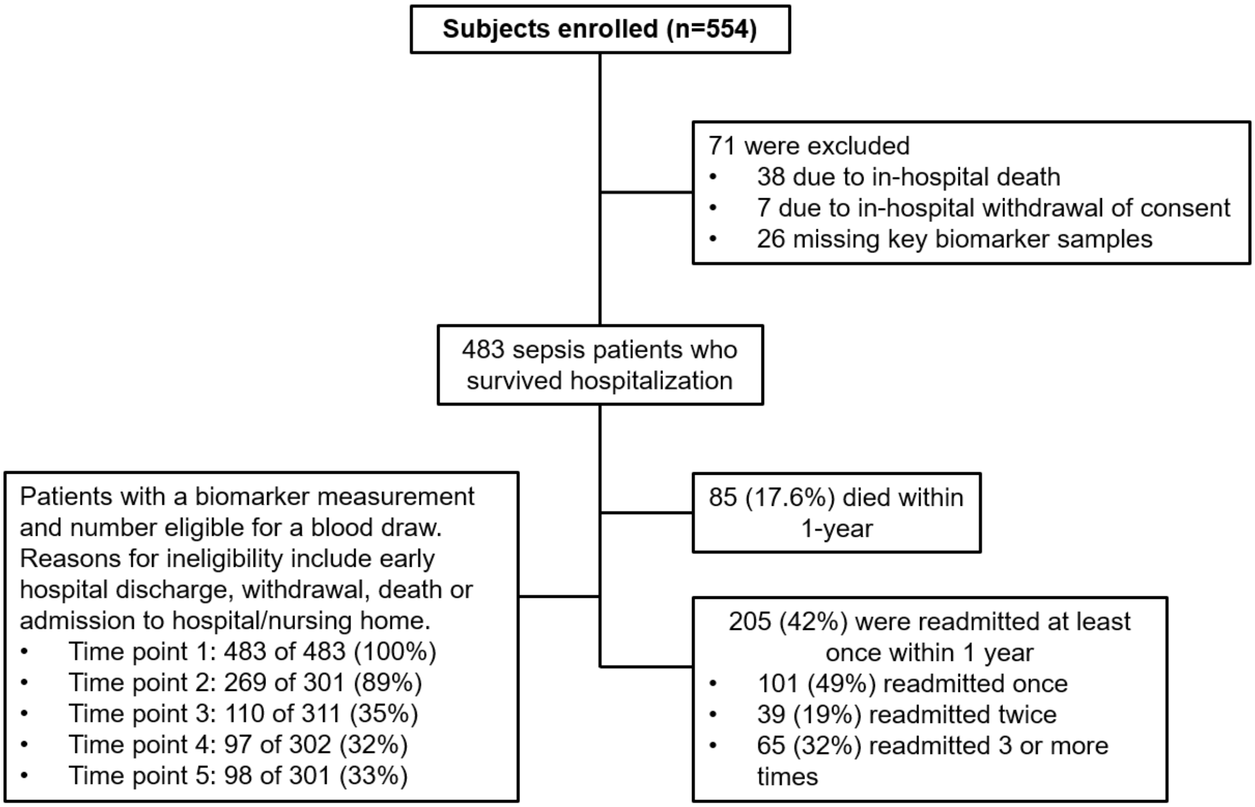

© 2019 Yende S et al. *JAMA Network Open.*

eFigure 2. Boxplots of Values for Markers of Endothelial Dysfunction, Hemostasis, and Oxidative Stress.

Data collected at each scheduled collection time point. Horizontal dotted lines represent the estimated 95th percentile of biomarker distribution among individuals without an infection.

© 2019 Yende S et al. *JAMA Network Open.*

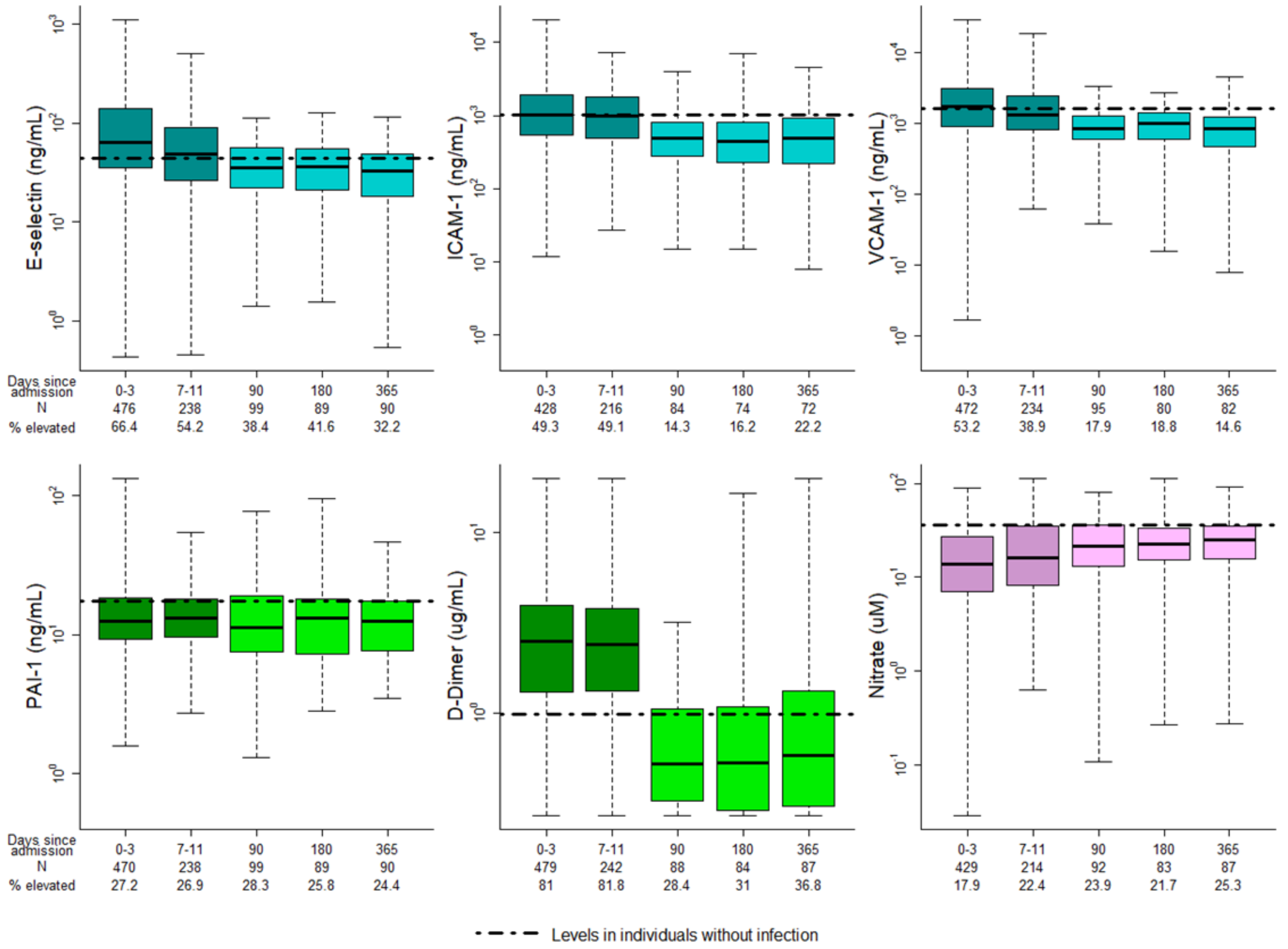

\

© 2019 Yende S et al. *JAMA Network Open.*

eFigure 3. Latent Longitudinal Trajectories From Non–Joint Latent-Class Mixture Models (LCMM) Using Biomarker Data From the First 13 Days or the In-hospital Period Only

Models with 1 to 4 mixture components were fit using data from each biomarker separately, and the models with the lowest BIC were chosen for plotting. The results show that for hs-CRP, PD-L1, e-selectin, ICAM-1, PAI-1 and Nitrate, >95% of the total sample was classified into a single latent class when only in-hospital data were used for classification. For IL-6 and VCAM-1, >80% had a single latent class. For D-dimer, 63%, had a single latent class.

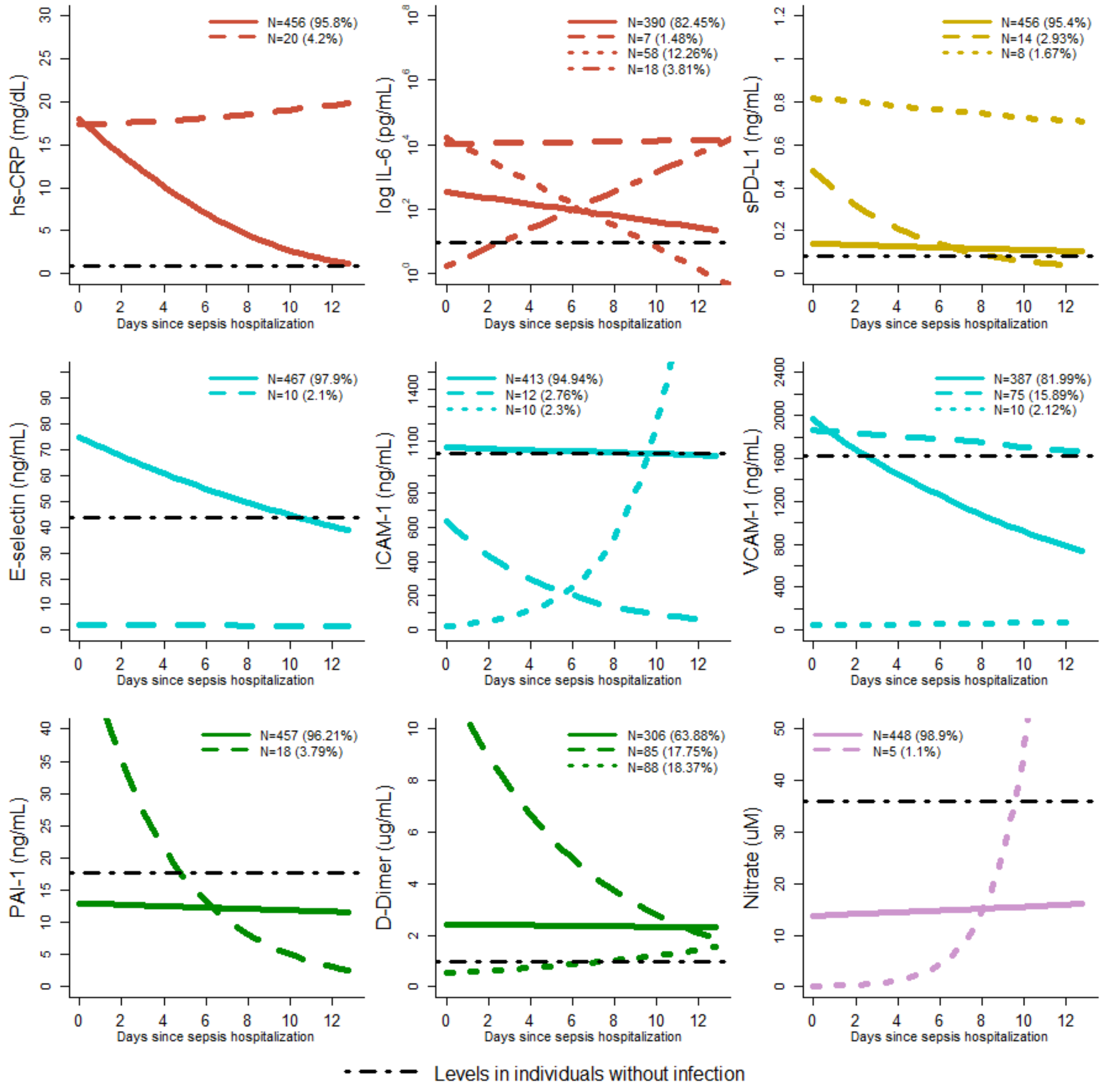

© 2019 Yende S et al. *JAMA Network Open.*

eFigure 4. Latent Trajectory Classes for Biomarkers of Hemostasis, Endothelial Dysfunction, and Oxidative Stress. Data estimated over 1 year using joint latent class mixture models (JLCMM). Horizontal dotted lines reprersent the estimated 95th percentile of biomarker distribution among individuals without an infection.

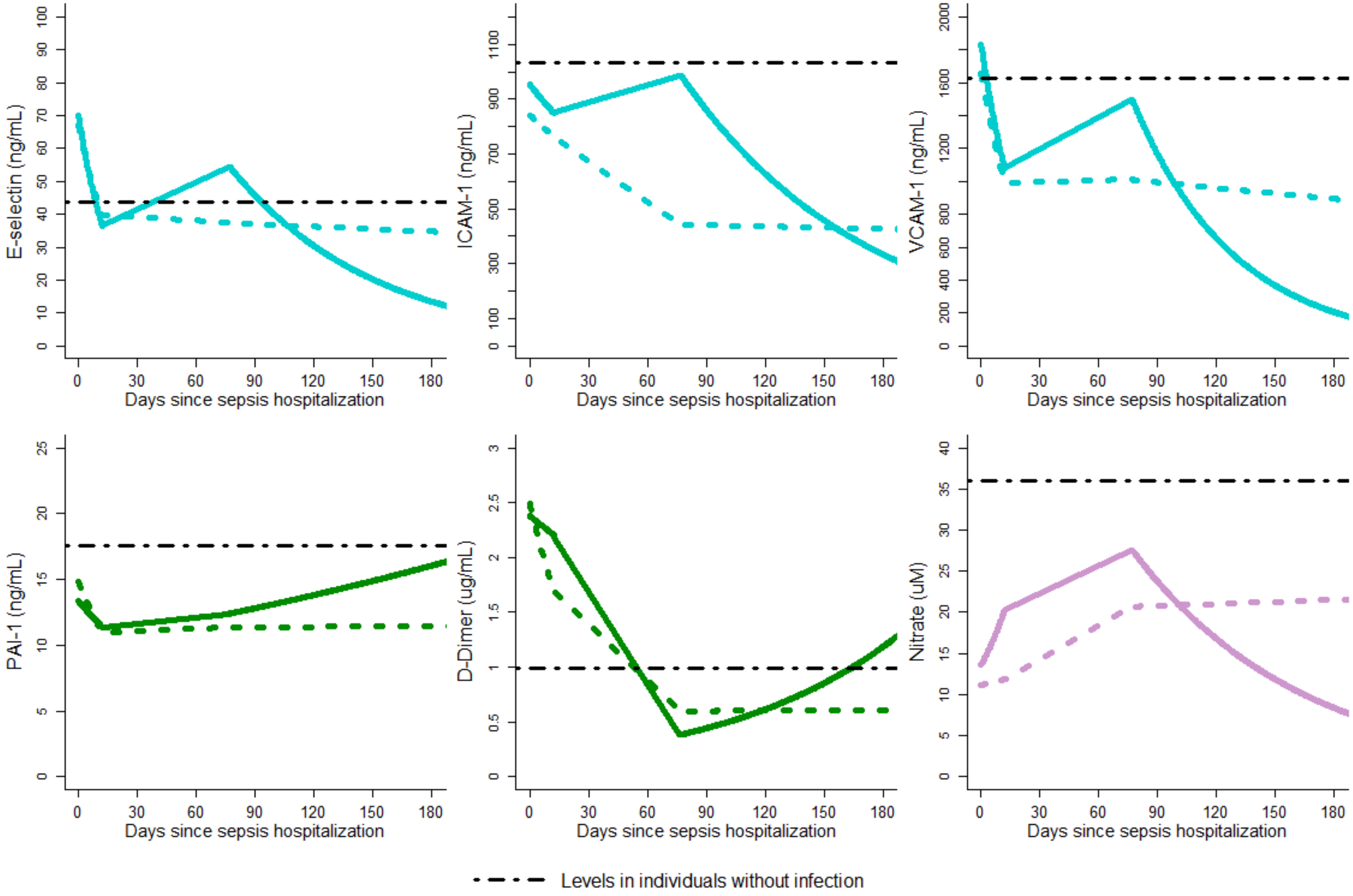

© 2019 Yende S et al. *JAMA Network Open.*

eFigure 5. JLCMM Model Results Using the 337 Patients With at Least 1 Postdischarge Biomarker Sample or Who Had Died or Were Readmitted Within 1 Year

Two-class models were fit to data for hs-CRP (left panel) and sPD-L1 (right panel). Green dotted lines are reference values in patients without sepsis or other inflammatory disordersl; black and red solid lines represent latent trajectories identified by the model..

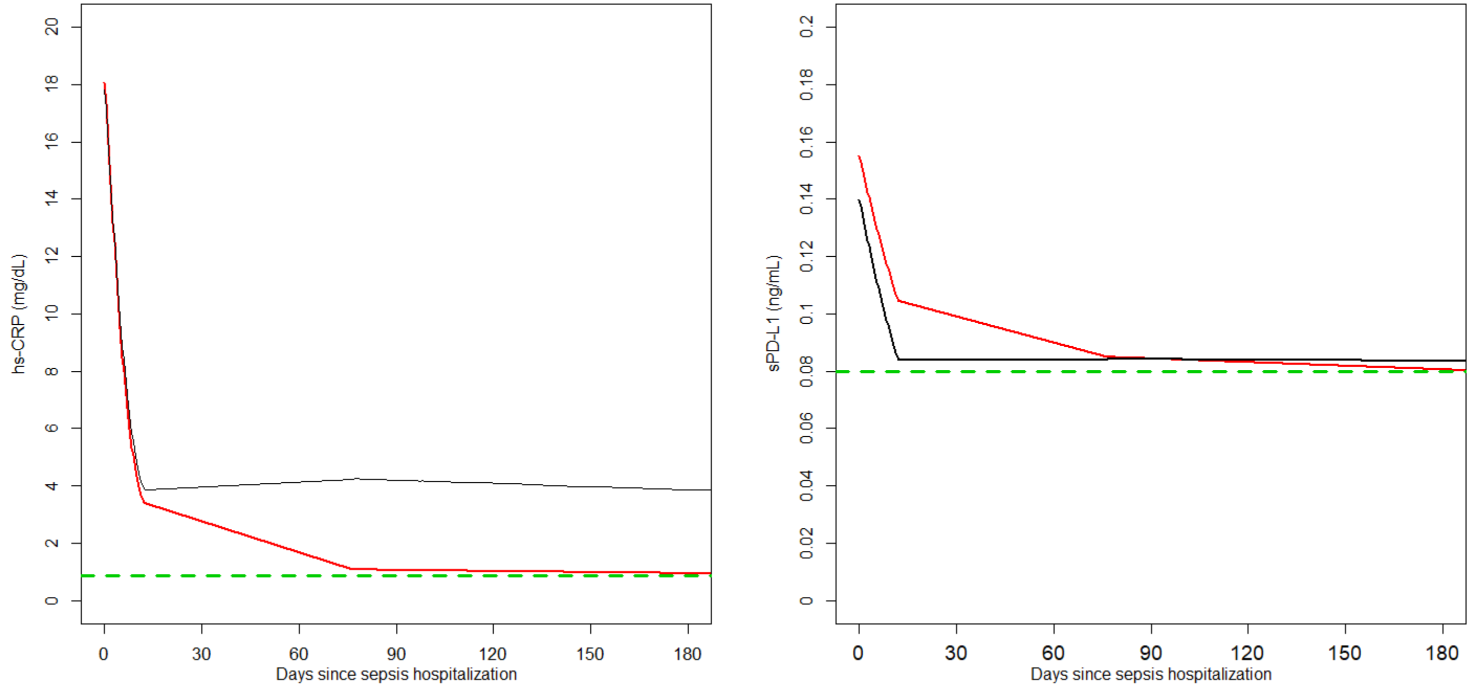

© 2019 Yende S et al. *JAMA Network Open.*

eFigure 6. Distribution of hs-CRP and PD-L1 for 4 Periods Stratified by Phenotypes Identified in the Primary JLCMM Analysis

Patients were included if they had at least one biomarker value collected during the period following discharge from the index hospitalization.

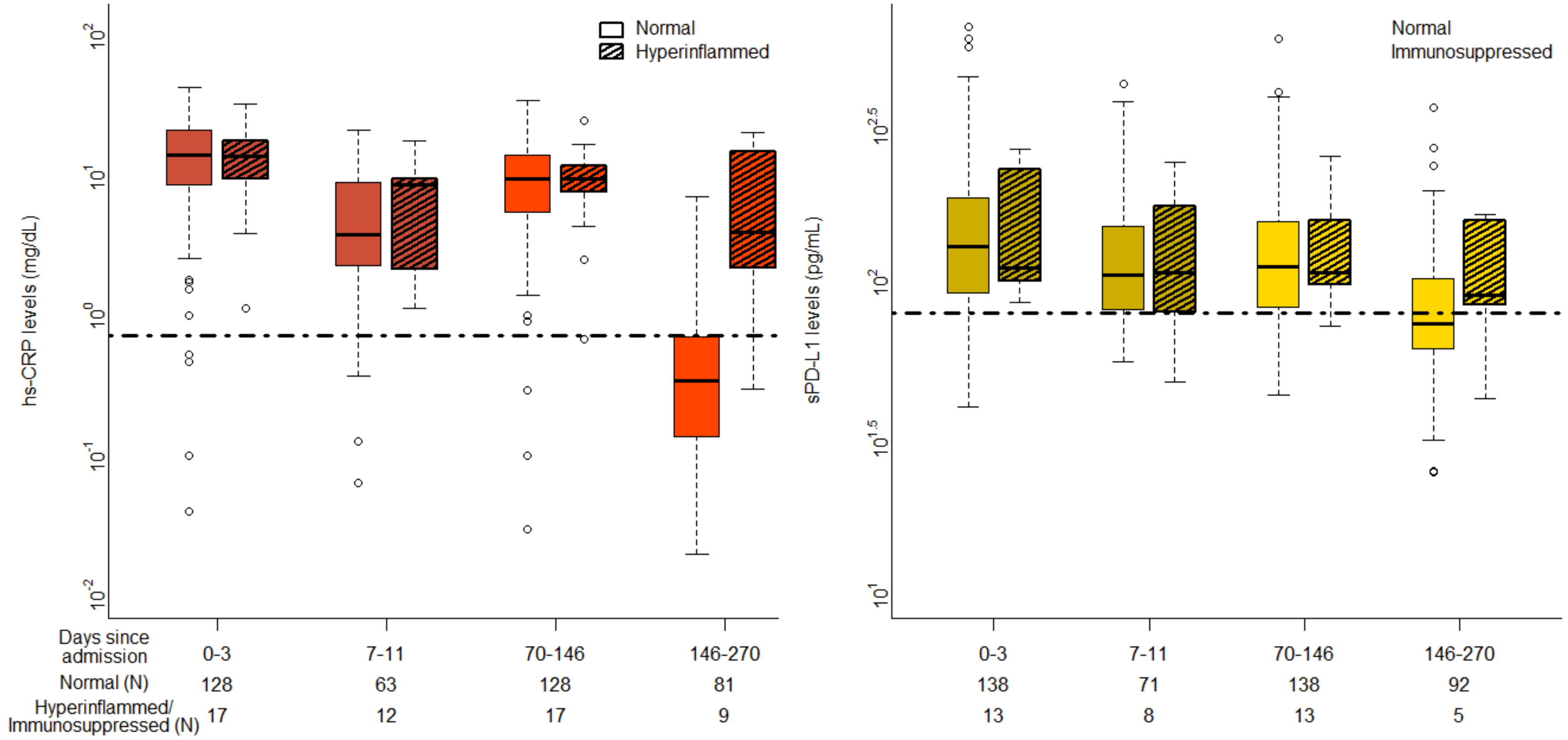

© 2019 Yende S et al. *JAMA Network Open.*

eFigure 7. Plots of hs-CRP and PD-L1 Levels Over Time, Among Patients With at Least 1 In-hospital and 1 Postdischarge Sample

Thick red and black lines represent latent trajectories identified in the joint latent class mixture model (JLCMM), while open circles joined by line segments represent observed data. Curves are shaded according to class membership. Model fit was optimised in each case using Bayes Information Criterion (BIC), which was minimized by the 2-class models fit to longitudinal biomarker and time to dropout data.

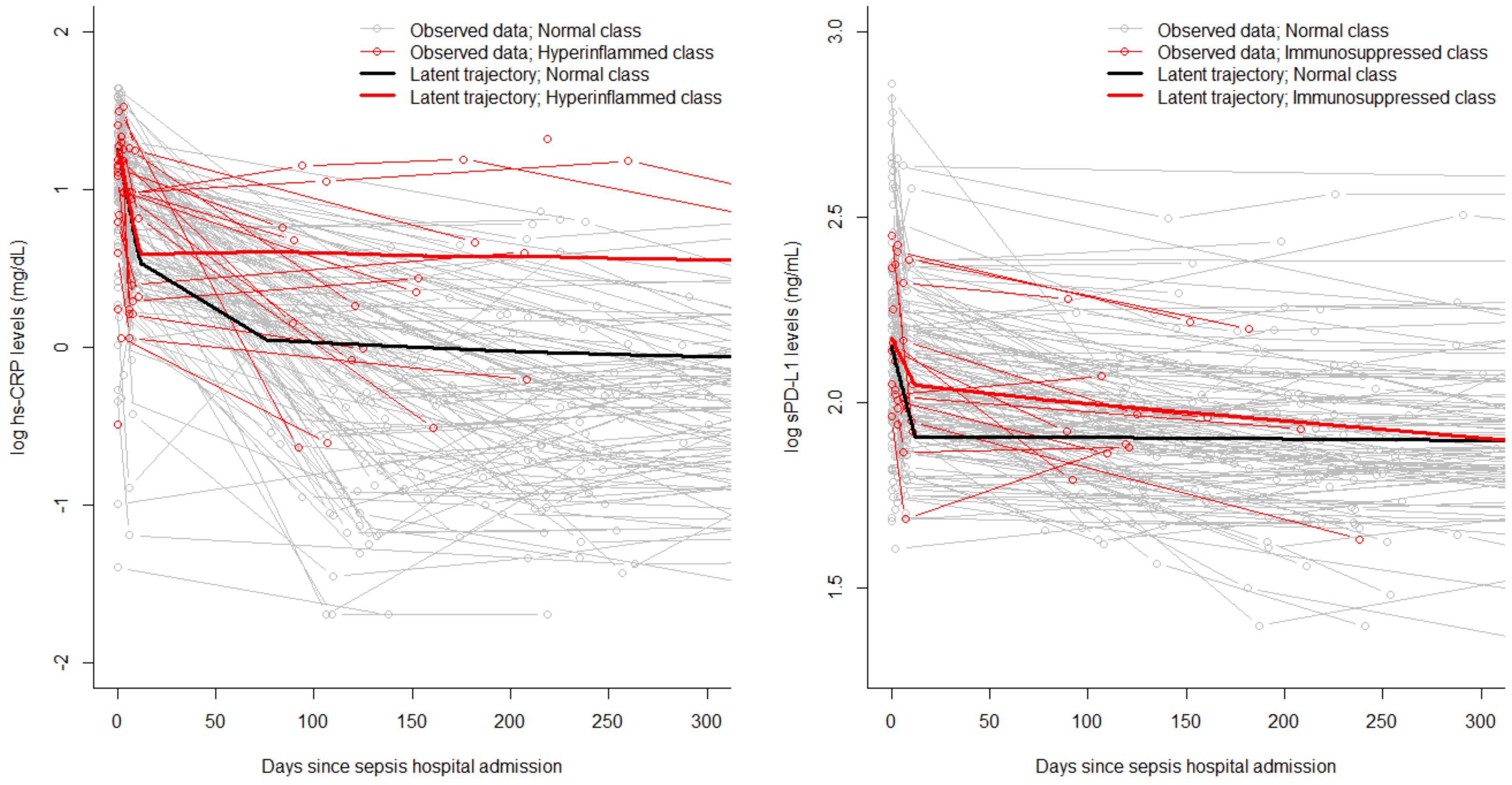

© 2019 Yende S et al. *JAMA Network Open.*

eFigure 8. Kaplan-Meier Plot of Mortality by Phenotype

This and other diagnostics assessing the proportionality of hazards between the two phenotypes suggested strongly that proportionality was violated. Because mortality was sparse in the normal phenotype, hazard ratios could not be estimated for the time period encompasing days 0 to 180 since hospitalization for sepsis. Therefore, the probability of mortality at 1 year was modeled using a logistic regression instead of hazard ratios using a Cox models.

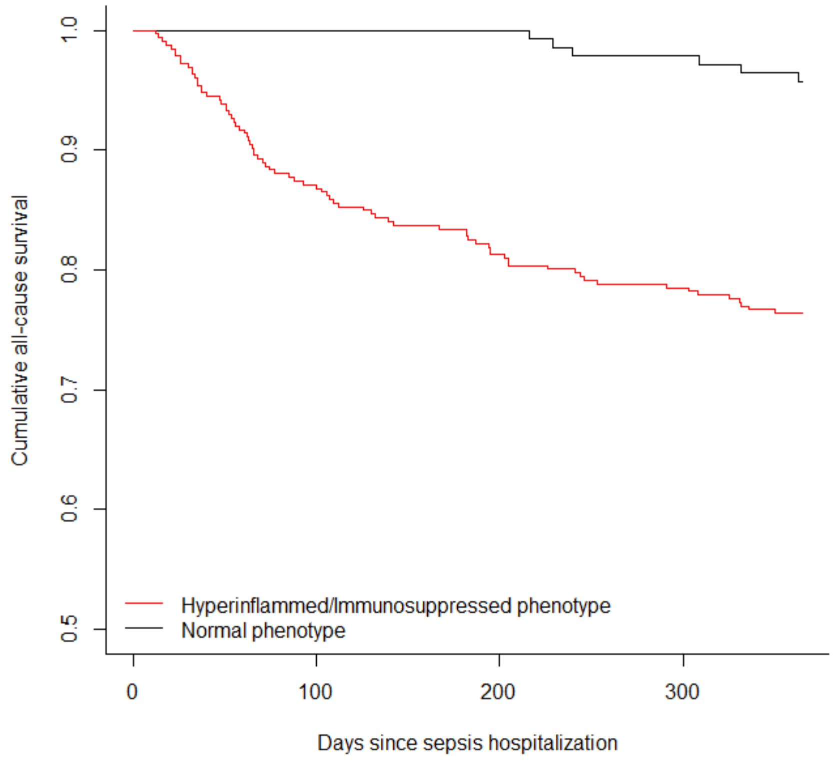

© 2019 Yende S et al. *JAMA Network Open.*

eFigure 9. Cumulative Incidence of Readmission and Mortality Stratified by Phenotype for 2 Hypothetical Patient Scenarios

The hyperinflammation/ immunosuppressed phenotype is represented by a solid line and red shading, and the normal phenotype by a dashed line and blue shading. Three event types are considered: all-cause readmission or mortality (panels A and B), readmission or mortality due to cardiovascular disease (panels C and D), and readmission or mortality due to cancer (panels E and F). Hypothetical patient #1 is a 50-year white male, without chronic diseases, hospitalized for sepsis due to pneumonia, an APACHE-II score of 8, and received mechanical ventilation. Patient #2 is a 70-year old female, with three chronic diseases, hospitalized for sepsis due to pneumonia, an APACHE II score of 16, and received vasopressors and dialysis. Cumulative incidence curves were estimated using Cox or Fine-Gray models and 95% pointwise confidence intervals were estimated via bootstrapping with 5000 resamples.

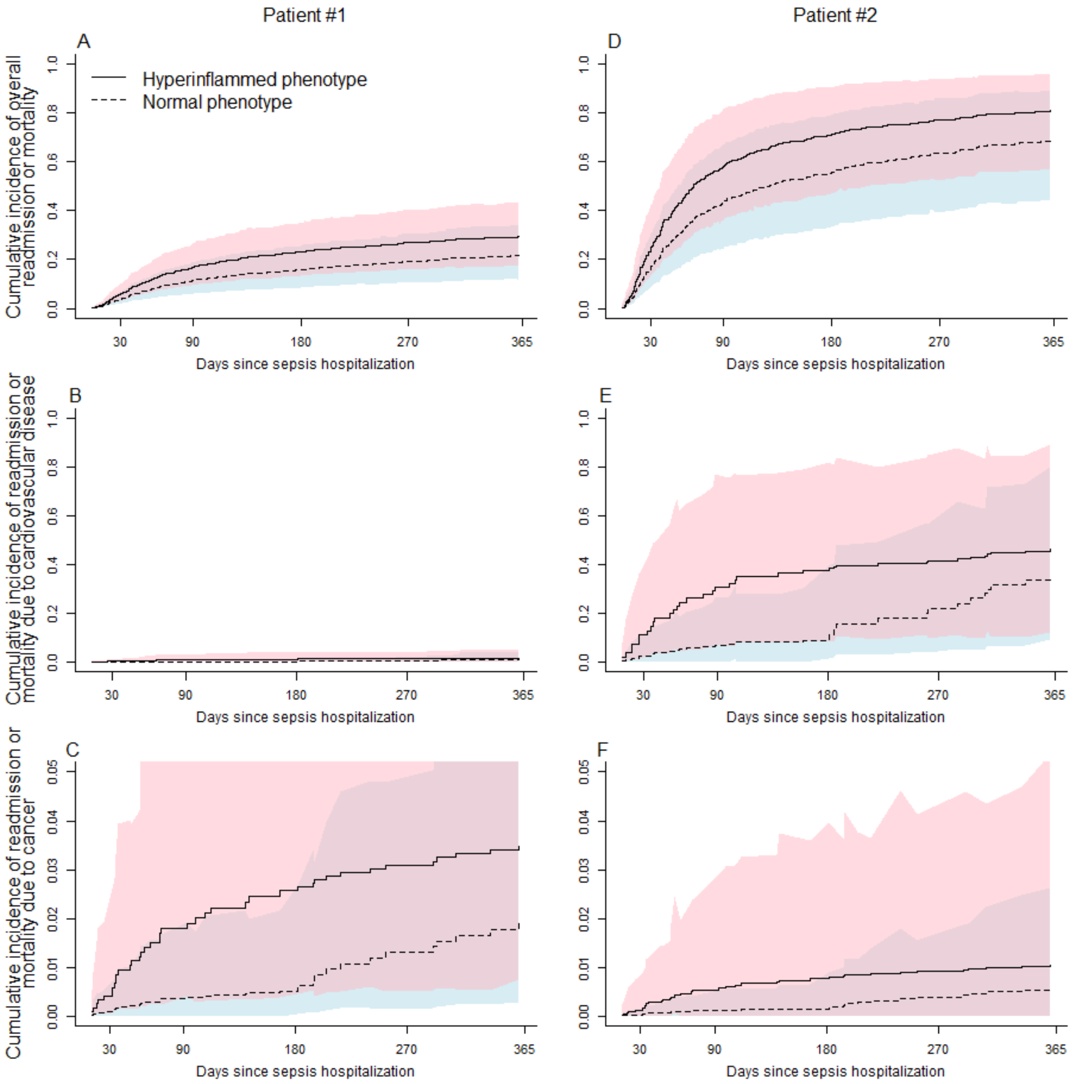

© 2019 Yende S et al. *JAMA Network Open.*

III. TABLES

eTable 1. Rationale for Selection of Reference value for Individuals Without an Infection

Biomarker	95th percentile reported by a clinical laboratory[5]	95th percentile reported by Univ of Pittsburgh or laboratory[6]	75th or 95th percentile reported in the literature[a]	Reference value in patients without an infection
IL-6 (pg/ml)	7 (n=817)	9.14 (n=10)	2.81 (n=40)[7]	9.14
hs-CRP (mg/dl)	0.2	0.88 (n=10)	0.55 (n=40)[7]	0.88
sPDL1 (ng/ml)	-	0.08 (n=35)[8,c]	0.07 (n=41)[9]	0.08
E-selectin (ng/ml)	-	43.62 (n=10)	32.92 (n=14)[10]	43.62
ICAM1 (ng/ml)	-	1032.11 (n=10)	328.6 (n=14)[11]	1032.11
VCAM1 (ng/ml)	-	1626.73 (n=10)	833.1 (n=140)[11]	1626.73
PAI-1 (ng/ml)	-	17.59 (n=10)	6.64 (n=9)[12]	17.59
D-dimer (ug/ml FEU)	0.99 (n=127)	0.64 (n=4)	0.38[13]	0.99
Nitrate (uM)	-	14.33 (n=7)	35.96 (n=14)[14]	35.96

[a]Some articles report 95th percentile ([a]), others report interquartile range ([b]) or mean plus standard deviation ([c]; SD). Where the 95th percentile was not available, we used the 75th percentile from the interquartile, or manually approximated the 95th percentile using the formula mean + 1.96*SD. For biomarkers where more than one upper percentile was reported in the literature, we selected the higher value.

eTable 2. Reason for Readmission Among Patients in the "Other Diagnoses" Category*

Cause of readmission	Frequency of readmissions due to other causes* (n=199)
Autoimmune	1
Benign prostatic hyperplasia	2
Coagulopathy	3
Endocrine	5
Fracture	2
Gastrointestinal	27
Hematology	10
Liver	16
Musculoskeletal	12
Neurology	8
Other cardiovascular	11
Overdose	2
Psychiatry	1
Pulmonary	13
Renal	21
Surgery unspecified	13
Transplant	3
Volume depletion and electrolyte abnormalities	11
Unknown**	38

*Patients not receiving diagnoses for infections (n=225), cardiovascular diseases (n=43), or cancers (n=20).

**Insufficient records to determine cause of readmission

eTable 3. Reason for Death Among Patients in the "Other Diagnoses" Category*

Cause of death	Frequency of deaths due to other causes* (n=25)
Trauma	1
Neurology	2
Pulmonary	2
Gastrointestinal	1
Renal	3
Unknown**	16

*Patients not receiving causes of death for cancer (n=32), cardiovascular disease (n=12), and infection (n=16).

**Insufficient records received to determine cause of death

© 2019 Yende S et al. *JAMA Network Open.*

eTable 4. Comparison of Clinical Characteristics Between Subjects With and Without Postdischarge Biomarker Measurement

© 2019 Yende S et al. *JAMA Network Open.*

Variables	No post discharge biomarker (n=318)	1 post-discharge biomarker measurement (n=67)	2 post-discharge biomarker measurement (n=56)	3 post-discharge biomarker measurement (n=42)
Demographics				
Age, median, mean (SD)	61, 60.1 (15.8)	59.1, 60 (16.2)	63.5, 61.8 (14.2)	62, 63.4 (10.1)
Sex, female, n (%)	143 (45)	28 (41.8)	22 (39.3)	25 (59.5)
Race, n (%)				
White	260 (81.8)	58 (86.6)	48 (85.7)	31 (73.8)
Black	43 (13.5)	7 (10.4)	6 (10.7)	9 (21.4)
Others	17 (5.3)	4 (6)	2 (3.6)	2 (4.8)
Chronic health conditions				
Charlson score, median, mean (SD)	1, 2.3 (2.5)	1, 2 (2.4)	1, 2.1 (2.4)	1, 1.7 (1.8)
Hypertension, n (%)	185 (58.7)	40 (59.7)	32 (58.2)	21 (50)
Myocardial infarction, n (%)	24 (7.6)	6 (9)	3 (5.5)	6 (14.3)
Congestive heart failure, n (%)	53 (16.8)	5 (7.6)	10 (18.2)	6 (14.3)
Cerebral vascular disease, n (%)	23 (7.3)	5 (7.5)	5 (9.1)	1 (2.4)
Peripheral vascular disease, n (%)	27 (8.6)	5 (7.5)	7 (12.7)	1 (2.4)

© 2019 Yende S et al. *JAMA Network Open.*

Chronic kidney disease, n (%)	68 (21.6)	14 (20.9)	11 (20)	8 (19.5)
Chronic dialysis, n (%)	23 (7.3)	4 (6.1)	2 (3.6)	4 (9.5)
Cirrhosis, n (%)	20 (6.3)	3 (4.5)	0 (0)	2 (4.8)
Chronic pulmonary disease, n (%)	69 (21.9)	13 (19.4)	16 (29.1)	12 (28.6)
Cancer	55 (98.2)	16 (94.1)	9 (100)	2 (66.7)
Medications, n (%)				
Anticoagulants	71 (22.5)	15 (22.4)	14 (25)	5 (11.9)
Statins	107 (34.1)	19 (28.4)	26 (46.4)	15 (35.7)
Steroids	59 (18.8)	9 (13.4)	15 (26.8)	6 (14.3)
Infection Site, n (%)				
Lung (Pneumonia)	65 (20.4)	8 (11.9)	16 (28.6)	13 (31)
Urosepsis	59 (18.6)	16 (23.9)	12 (21.4)	7 (16.7)
Intra-abdominal	46 (14.5)	16 (23.9)	12 (21.4)	10 (23.8)
Skin and soft-tissue	38 (11.9)	5 (7.5)	2 (3.6)	3 (7.1)
Catheter-related	13 (4.1)	3 (4.5)	2 (3.6)	1 (2.4)
Central nervous system	0 (0)	1 (1.5)	0 (0)	0 (0)
Endocarditis	6 (1.9)	1 (1.5)	0 (0)	0 (0)

© 2019 Yende S et al. *JAMA Network Open.*

Illness severity, median, mean (SD)				
APACHE[†] II Score	11, 12.5 (6)	12, 13.2 (5.9)	11, 11.5 (5.8)	12, 13.9 (7)
Total SOFA[§] Score	4, 4 (2.9)	4, 4.6 (3.3)	3.5, 3.9 (2.7)	4, 5.2 (3.6)
Cardiovascular SOFA	1, 0.7 (0.8)	1, 0.8 (0.9)	1, 0.8 (0.9)	1, 1.1 (1.2)
Central nervous system SOFA	0, 0.4 (0.9)	0, 0.5 (1.1)	0, 0.3 (0.9)	0, 0.6 (1.1)
Coagulation SOFA	0, 0.6 (1)	0, 0.6 (1)	0, 0.5 (0.8)	0, 0.6 (0.8)
Liver SOFA	0, 0.4 (0.9)	0, 0.5 (0.9)	0, 0.5 (0.9)	0, 0.4 (0.7)
Renal SOFA	1, 1.4 (1.3)	1, 1.4 (1.3)	1, 1.2 (1.3)	1, 1.5 (1.3)
Respiration SOFA	0, 0.5 (1.1)	0, 0.7 (1.2)	0, 0.6 (1.2)	0, 0.9 (1.4)
Organ support, n (%)				
Mechanical Ventilation	54 (17)	15 (22.4)	10 (17.9)	12 (28.6)
Vasopressor Use	112 (35.2)	33 (49.3)	27 (48.2)	22 (52.4)
Dialysis	32 (10.1)	6 (9)	2 (3.6)	2 (4.8)
Hospital stay in days, mean (sd, median)	8, 10.9 (10.4)	6, 11.8 (15.6)	7, 8.8 (7.2)	7, 9 (5.9)

© 2019 Yende S et al. *JAMA Network Open.*

eTable 5. Observed Distribution of Circulating Biomarkers by Time Point

Biomarker	Time point	Minimum	25th percentile	Median	Mean	75th percentile	Maximum
hs-CRP	1	0.04	8.81	14.61	16.04	21.89	48
	2	0.06	2.43	4.8	7.4	10.07	40.47
	3	0.02	0.13	0.41	1.17	0.94	14.17
	4	0.02	0.16	0.48	1.61	1.23	20.93
	5	0.02	0.16	0.39	0.86	0.92	6.95
IL-6	1	0.75	102.99	336.2	3606.38	1308.38	101886.9
	2	0.54	41.69	109.48	1106.11	295.56	26738
	3	0.74	8.29	33.11	80.14	68.87	991.6
	4	1.59	2.96	21.16	69.05	54.76	940.63
	5	1.46	5.24	22.62	935.58	60.09	26738
PD-L1	1	11.62	93.45	135.17	186.42	210.97	1678.75
	2	6.05	92.26	120.06	168.37	186.01	1678.75
	3	31.57	62.8	77.32	89.31	105.32	314.4
	4	24.97	62.19	75.47	91.3	104.48	365.65
	5	19.95	62.46	77.86	97.61	107.56	401.53
E-selectin	1	0.42	35.31	63.27	115.82	139.13	1117
	2	0.45	26.3	47.28	69.57	84.33	503.98
	3	1.41	22.45	35.23	41.97	57.22	113.79
	4	1.54	21.08	36.96	42.87	56.47	126.75
	5	0.53	18.21	32.52	38.01	49.07	239.23

© 2019 Yende S et al. *JAMA Network Open.*

ICAM-1	1	12.01	543.96	1024.6	1542.49	1934.22	20436
	2	27.63	490.21	1037.25	1378.52	1866.02	9027
	3	14.98	286.54	490.12	667.48	802.23	4067.5
	4	14.98	227.67	464.58	750.28	809.54	7004.2
	5	8	218.24	486.01	702.85	942.15	4523.1
VCAM-1	1	1.68	985.56	1809.6	3104.66	3497.3	41512
	2	62.72	831.67	1419.55	2513.11	2637.7	40651
	3	39.08	607.91	854.97	1021.24	1277.1	3377
	4	15.84	620.17	1031.9	1099.03	1451.6	2744.2
	5	8.01	478.26	848.55	987.72	1230.15	4540.7
PAI-1	1	1.6	9.4	13.01	16.82	18.81	133.84
	2	2.71	9.64	13.57	15.98	18.44	75.67
	3	1.31	7.58	11.43	14.85	19.23	77.66
	4	2.84	7.95	13.34	15.5	18.87	95.7
	5	3.47	8.38	12.77	14.09	17.4	46.11
D-dimer	1	0.27	1.33	2.54	4.13	4.35	20
	2	0.27	1.38	2.61	3.98	3.91	20
	3	0.27	0.34	0.52	0.77	1.03	3.2
	4	0.27	0.28	0.51	1.05	1.04	16.53
	5	0.27	0.32	0.61	1.79	1.33	20
Nitrate	1	0	4.9	12.25	19.04	26.47	100.28
	2	0	5.96	13.07	21.09	30.09	113.59
	3	0	9.04	19.29	22.14	32.86	80.51

© 2019 Yende S et al. *JAMA Network Open.*

	4	0	9.78	20.6	23.77	32.56	113.6
	5	0	11.91	21.91	24.07	32.01	91.94

© 2019 Yende S et al. *JAMA Network Open.*

eTable 6. Distribution of Combined Phenotypes* Among Patients in 6 Different Subgroups

Phenotypes were derived from the primary analysis in all N=483 patients.

Patient subset	Hyperinflammatory / immunosuppressed phenotype	Normal phenotype	Only hyperinflamed phenotype	Only immunosuppressed phenotype
	Frequency (%)	Frequency (%)	Frequency (%)	Frequency (%)
Younger than 50 years*	75 (72.1)	27 (26.0)	2 (1.9)	0 (0)
No chronic diseases history*	98 (65.8)	50 (33.6)	0 (0)	1 (0.7)
No cancer history or readmission*	258 (68.1)	113 (29.8)	6 (1.6)	2 (0.5)
Respiratory source of infection*	63 (67.7)	30 (32.3)	0 (0.0)	0 (0.0)
Non-respiratory source of infection*	264 (68.8)	111 (28.9)	7 (1.8)	2 (0.5)
Assessed for organ failure within 24 hrs*	208 (70.5)	81 (27.5)	5 (1.7)	1 (0.3)
Contributed at least 1 post-discharge sample, or had died or readmitted within 365 days**	188 (56.3)	117 (35.0)	2 (1.0)	27 (8.1)

* Phenotypes generated from the primary analysis among all N=483 patients

** Phenotypes generated from the secondary analysis using patients in this subset (N=337)

© 2019 Yende S et al. *JAMA Network Open.*

eTable 7. Association Between Phenotypes and Long-term Outcomes Among Individuals With Posterior Probability of Membership Into a Phenotype Greater Than 80% in the Primary Analysis

© 2019 Yende S et al. *JAMA Network Open.*

Variables	Hyperinflammatory/ immunosuppressed phenotype	Normal phenotype	Adjusted**** OR, HR, or SHR (95% confidence intervals)	P-value
	No. of events/no. at risk (%)	No. of events/no. at risk (%)		
All-cause 1-year mortality	73 / 297 (24.6)	4 / 134 (3.0)	13.30 (4.48, 39.5)	<0.0001
All-cause readmission or death				
0-180 d	129 / 297 (43.4)	48 / 134 (35.8)	1.41 (1.01, 1.97)	0.04
180-365 d	27 / 168 (16.1)	12 / 86 (14.0)	1.35 (0.68, 2.66)	0.39
Readmission or death due to infection				
0-180 d	68 / 297 (22.9)	27 / 134 (20.1)	1.22 (0.777, 1.901)	0.39
180-365 d	20 / 200 (10.0)	4 / 107 (3.7)	2.32 (0.78, 6.86)	0.13
Readmission or death due to cardiovascular disease				
0-180 d	20 / 297 (6.7)	2 / 134 (1.5)	4.75 (1.10, 20.48)	0.04
180-365 d	5 / 233 (2.1)	6 / 132 (4.5)	0.43 (0.13, 1.43)	0.17
Readmission or death due to cancer				
0-180 d	24 / 297 (8.1)	2 / 134 (1.5)	4.84 (1.17, 19.93)	0.03
180-365 d	6 / 243 (2.5)	3 / 132 (2.3)	0.97 (0.23, 4.12)	0.97

* Odds ratios estimated using logistic regression model

** Hazard ratios estimated using Cox model

*** Subdistribution hazard ratios estimated using Fine-Gray model

**** Covariates included age, sex, race, Charlson score, APACHE-II score, infection site, mechanical ventilation, vasopressor use, and dialysis.

© 2019 Yende S et al. *JAMA Network Open.*

eTable 8. Association Between Phenotypes and Long-term Outcomes Among Individuals in the Sensitivity Analysis Among Subjects Who Had at Least 1 Postdischarge Biomarker Sample or Who Died or Were Readmitted Within 1 Year (N=337)

© 2019 Yende S et al. *JAMA Network Open.*

Variables	Hyperinflammatory/ immunosuppressed phenotype	Normal phenotype	Adjusted**** OR, HR, or SHR (95% confidence intervals)	P-value
	No. of events/no. at risk (%)	No. of events/no. at risk (%)		
All-cause 1-year mortality	77 / 188 (41.0)	3 / 117 (2.6)	37.29 (10.48, 132.64)	<0.0001
All-cause readmission or death				
0-180 d	148 / 188 (78.7)	39 / 117 (33.3)	4.06 (2.82, 5.85)	<0.0001
180-365 d	30 / 40 (75.0)	10 / 78 (12.8)	14.7 (7.55, 28.63)	<0.0001
Readmission or death due to infection				
0-180 d	80 / 188 (42.6)	21 / 117 (18.0)	3.08 (1.9, 5)	<0.0001
180-365 d	22 / 78 (28.2)	2 / 96 (2.1)	11.63 (2.75, 49.14)	0.0008
Readmission or death due to cardiovascular disease				
0-180 d	22 / 188 (11.7)	2 / 117 (1.7)	8.02 (1.74, 36.94)	0.01
180-365 d	6 / 120 (5.0)	4 / 115 (3.5)	1.25 (0.33, 4.67)	0.74
Readmission or death due to cancer				
0-180 d	25 / 188 (13.3)	1 / 117 (0.9)	13.22 (1.65, 105.77)	0.02
180-365 d	7 / 131 (5.0)	3 / 116 (2.6)	1.67 (0.43, 6.5)	0.46

* Odds ratios estimated using logistic regression model

** Hazard ratios estimated using Cox model

*** Subdistribution hazard ratios estimated using Fine-Gray model

**** Covariates included age, sex, race, Charlson score, APACHE-II score, infection site, mechanical ventilation, vasopressor use, and dialysis.

© 2019 Yende S et al. *JAMA Network Open.*

eTable 9. Association Between Phenotypes as Determined by the Primary Analysis Joint Models and Long-term Outcomes Among 5 Subsets of Individuals With Specific Characteristics

© 2019 Yende S et al. *JAMA Network Open.*

Variables	Hyperinflammatory/ immunosuppressed phenotype	Normal phenotype	Adjusted*** OR, HR, (95% confidence intervals)	P-value
	No. of events/no. at risk (%)	No. of events/no. at risk (%)		
Younger than 50 years				
All-cause 1-year mortality	7 / 75 (9.3)	0 / 27 (0.0)	n/a	n/a
All-cause readmission or death				
0-180 d	23 / 75 (30.7)	10 / 27 (37.0)	0.85 (0.36, 2.01)	0.71
180-365 d	4 / 52 (7.7)	3 / 17 (17.6)	0.56 (0.11, 2.71)	0.47
No chronic diseases history				
All-cause 1-year mortality	9 / 98 (9.2)	1 / 50 (2.0)	7.75 (0.73, 82.64)	0.09
All-cause readmission or death				
0-180 d	29 / 98 (29.6)	14 / 50 (28.0)	1.11 (0.58, 2.11)	0.76
180-365 d	4 / 69 (5.8)	8 / 36 (22.2)	0.24 (0.07, 0.80)	0.02
No cancer				
All-cause 1-year mortality	41 / 258 (15.9)	2 / 113 (1.8)	12.03 (2.80, 51.7)	0.0008
All-cause readmission or death				
0-180 d	105 / 258 (40.7)	39 / 113 (34.5)	1.35 (0.93, 1.95)	0.11
180-365 d	20 / 153 (13.1)	9 / 74 (12.2)	1.15 (0.53, 2.53)	0.72
Respiratory source of infection				
All-cause 1-year mortality	19 / 63 (30.2)	2 / 30 (6.7)	7.67 (1.47, 40.17)	0.02
All-cause readmission or death				
0-180 d	33 / 63 (52.4)	11 / 30 (36.7)	1.89 (0.99, 3.59)	0.05
180-365 d	4 / 30 (13.3)	5 / 19 (26.3)	0.52 (0.14, 1.89)	0.32
Non-respiratory source of infection				

© 2019 Yende S et al. *JAMA Network Open.*

All-cause 1-year mortality	58 / 264 (22.0)	4 / 111 (3.6)	8.55 (2.95, 24.75)	<0.0001
All-cause readmission or death				
0-180 d	112 / 264 (42.4)	37 / 111 (33.3)	1.56 (1.06, 2.28)	0.02
180-365 d	26 / 152 (17.1)	10 / 74 (13.5)	1.55 (0.75, 3.2)	0.24
Assessed for organ failure within 24 hrs				
All-cause 1-year mortality	53 / 208 (25.5)	5 / 81 (6.2)	5.64 (2.11, 15.05)	0.0006
All-cause readmission or death				
0-180 d	93 / 208 (44.7)	30 / 81 (37.0)	1.43 (0.95, 2.13)	0.09
180-365 d	24 / 115 (20.9)	10 / 51 (19.6)	1.20 (0.58, 2.48)	0.62

* Odds ratios estimated using logistic regression model

** Hazard ratios estimated using Cox model

*** Covariates included age, sex, race, Charlson score, APACHE-II score, infection site, mechanical ventilation, vasopressor use, and dialysis

© 2019 Yende S et al. *JAMA Network Open.*

eTable 10. Reasons for Readmission by Phenotype*

Cause of readmission	Hyperinflammed / Immunosuppressed phenotype, (n=297)	Normal phenotype (n=134)
Infection	163	61
Cardiovascular diseases	27	12
Cancers	16	4
Other diagnoses	119	67

* Numbers in each row may not add to the total number of readmissions for each condition if some patients were not able to be assigned to one of the 2 most common phenotypes.

eTable 11. Reasons for Death Stratified by Phenotype

Cause of death	Hyperinflammed / Immunosuppressed phenotype (n=297)	Normal phenotype (n=134)
Cancer	27	4
Cardiovascular	11	1
COPD	2	0
Other diagnoses	24	1

© 2019 Yende S et al. *JAMA Network Open.*

eReferences

1. Ives DG, Fitzpatrick AL, Bild DE, et al. Surveillance and ascertainment of cardiovascular events. The Cardiovascular Health Study. *Ann Epidemiol.* 1995;5(4):278-285.

2. Proust-Lima C, Letenneur L, Jacqmin-Gadda H. A nonlinear latent class model for joint analysis of multivariate longitudinal data and a binary outcome. *Stat Med.* 2007;26(10):2229-2245.

3. Yende S, D'Angelo G, Kellum JA, et al. Inflammatory Markers at Hospital Discharge Predict Subsequent Mortality after Pneumonia and Sepsis. *American Journal of Respiratory and Critical Care Medicine.* 2008;177(11):1242-1247.

4. Corrales-Medina VF, Alvarez KN, Weissfeld LA, et al. Association between hospitalization for pneumonia and subsequent risk of cardiovascular disease. *Jama.* 2015;313(3):264-274.

5. Laboratory MCLhwmct-cCaIoUoPMCC. Mayo Clinic Laboratory (https://www.mayomedicallaboratories.com/test-catalog/Clinical+and+Interpretive/40936) or University of Pittsburgh Medical Center Clinical Laboratory.

6. Palmer OMP, Carter M, Chang C-CH, et al. Effects of Transport Temperature on the Stability of Inflammatory, Hemostasis, Endothelial Function, and Oxidative Stress Plasma Biomarker Concentrations. *Shock (Augusta, Ga).* 2017;47(6):715-719.

7. Hubbard RE, O'Mahony MS, Savva GM, Calver BL, Woodhouse KW. Inflammation and frailty measures in older people. *J Cell Mol Med.* 2009;13(9B):3103-3109.

8. Stortz JA, Murphy TJ, Raymond SL, et al. Evidence for Persistent Immune Suppression in Patients Who Develop Chronic Critical Illness After Sepsis. *Shock.* 2018;49(3):249-258.

9. Kruger S, Legenstein ML, Rosgen V, et al. Serum levels of soluble programmed death protein 1 (sPD-1) and soluble programmed death ligand 1 (sPD-L1) in advanced pancreatic cancer. *Oncoimmunology.* 2017;6(5):e1310358.

10. Semaan HB, Gurbel PA, Anderson JL, et al. Soluble VCAM-1 and E-selectin, but not ICAM-1 discriminate endothelial injury in patients with documented coronary artery disease. *Cardiology.* 2000;93(1-2):7-10.

11. Pradhan AD, Rifai N, Ridker PM. Soluble intercellular adhesion molecule-1, soluble vascular adhesion molecule-1, and the development of symptomatic peripheral arterial disease in men. *Circulation.* 2002;106(7):820-825.

© 2019 Yende S et al. *JAMA Network Open.*

12. Patel N, Sundaram N, Yang M, Madigan C, Kalra VK, Malik P. Placenta growth factor (PlGF), a novel inducer of plasminogen activator inhibitor-1 (PAI-1) in sickle cell disease (SCD). *J Biol Chem.* 2010;285(22):16713-16722.

13. Nomura H, Wada H, Mizuno T, et al. Negative predictive value of D-dimer for diagnosis of venous thromboembolism. *Int J Hematol.* 2008;87(3):250-255.

14. Ochoa JB, Udekwu AO, Billiar TR, et al. Nitrogen oxide levels in patients after trauma and during sepsis. *Ann Surg.* 1991;214(5):621-626.

© 2019 Yende S et al. *JAMA Network Open.*
